# Challenges and opportunities of hydrothermal carbonisation in the UK; case study in Chirnside

**DOI:** 10.1039/d1ra06736b

**Published:** 2021-10-27

**Authors:** Eloise Bevan, Jile Fu, Mauro Luberti, Ying Zheng

**Affiliations:** Institute for Materials and Processes, School of Engineering, The University of Edinburgh Edinburgh EH9 3FB UK bevan.eloise@gmail.com; Department of Chemical and Biochemical Engineering, Western University London Ontario N6A 5B9 Canada

## Abstract

The latest research and development in hydrothermal carbonisation (HTC) processes are reviewed and the feasibility of application to small towns in the UK is assessed. The HTC process designed in this report is theoretically evaluated for the biodegradable municipal waste and sewage waste produced by the small town of Chirnside, in the Scottish Borders. Calculation of mass and energy balances of the process are carried out alongside the evaluation of challenges and environmental, social and economic opportunities presented. The hypothetical HTC plant is capable of processing 267.14 t per year of food waste and 105.12 t per year of faecal sludge produced by Chirnsides estimated 2250 residents in 2041. The plant would be capable of producing 99.08 t per year of hydrochar with an estimated total energy content of 540.26 MWh per year. When used in a Biomass Combined Heat and Power Plant, the hydrochar would be capable of supplying Chirnsides residents with 0.71% and 3.43% of its domestic thermal energy demand and domestic electrical energy demand in 2041, respectively. Both the expected opportunities and challenges for the application of HTC are discussed, shedding light on the associated research in regards to this sustainable technology.

## Abstract 2

The latest research and development in hydrothermal carbonisation (HTC) processes are reviewed and the feasibility of application to small towns in the UK is assessed.

## Graphical abstract

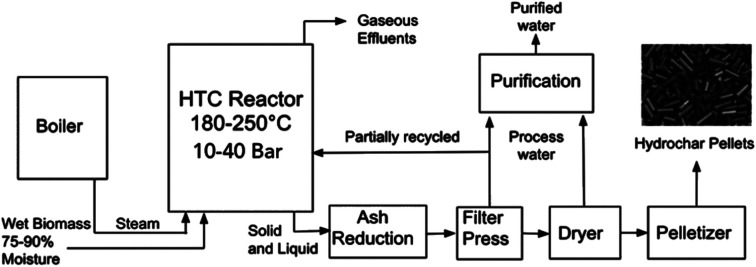

## 1. Introduction

Fossil fuels, as an energy source, accounted for 84% of the world's total energy consumption in 2019.^1^ This demonstrates a reduction in consumption when compared to the start of the industrial revolution when alternative energy sources were scarce. However, this reliance on fossil fuels in the 21^st^ century is unsustainable as the world's reserves are limited and are continually depleting. This depletion of reserves demonstrates the need for alternative energy sources and that the investment into the development of their technology is paramount for sustainable development.

In order to minimise the reliability on fossil-based energy sources, there is a requirement for the continuation of research into technology that drives the renewable energy sector. One such renewable resource includes biomass, the official term denoted to organic matter that can be optimised as an energy source. Although biomass technologies are relatively new to modern societies, the energy that can be harvested from biomass has been used by humankind as a heat source since the dawn in our discovery of fire, approximately 4–500 000 years ago.^2^ Despite our daily and world-wide consumption of this fuel it has only been until the 21st century that large scale, industrial harvesting of this energy is being introduced into countries worldwide. Harvesting of energy from biomass has been coined bioenergy, in which the state of matter defines three broad categories of biofuels: solid biomass (*e.g.*, wood, harvesting residues, pellets), liquid biofuel (*e.g.* bioethanol, biodiesel) and gaseous biofuels (*e.g.*, biogas). Comparing to the world's fossil fuel consumption, bioenergy contributes approximately 10% of the world's total energy production and is the largest renewable energy source that is presently used.^1^ The following shares of this contribution by region have been estimated as the following; North America (44.1%), South and Central America (28.7%), Europe and Eurasia (16.5%), Asia Pacific (10.6%), Middle East (∼0%) and Africa (∼0%).^3^

As global energy demands grow exponentially with time, the number of research projects into various, large-scale biomass processes increases.^4^ Besides the traditional thermal conversion of biomass (combustion), there are currently three main process technologies currently available: bio-chemical, thermo-chemical and physio-chemical. Bio-chemical conversion encompasses two primary process options: anaerobic digestion (to biogas) and fermentation (to ethanol) where enzymes or microorganisms break down the biomass into liquid fuels. Physio-chemical conversion consists principally of extraction (with esterification) where oilseeds are crushed to extract oil. Thermo-chemical conversion processes include gasification, pyrolysis and hydrothermal carbonisation (wet pyrolysis).^5^

The main focus of this review is hydrothermal carbonisation (HTC), which was first studied over a century ago by Nobel Prize winner Friedrich Bergius (1913).^6^ This technology presents a relatively new, renewable and innovative process that has only started to be applied on an industrial scale. HTC processing of biomass is similar to the previously mentioned thermo-chemical processes, as they are all operated by exposing organic substrates to elevated temperatures; however, contrary to the other biomass processes, HTC operates at an elevated pressure and is capable of processing feeds with a moisture content of 75–90%.^7^ This includes (but not limited to) agricultural waste such as alongside horse manure,^11^ municipal waste, organic waste from the industrial food sector, sewage sludge,^7^ green waste^12^ to fiber sludge derived from the paper industry.^13^ The final product of this biomass reformation process is a carbon-based solid, referred to as ‘hydrochar’. Due to the compactness of nutrients in the hydrochar pellets, which can be produced without binders or expensive drying procedures,^14^ they can be applied in agricultural practices for soil amendment.^15^ the more beneficial application which is igniting the interest of researchers worldwide is its ability to act as a neutral combustible being an energy-dense source of carbon. There are various wet biomass sources for hydrochar production the calorific value and quality of hydrochar pellets is dependent on the biomass feedstock.^16,17^ In addition, as the severity of carbonisation increases (higher temperature, longer residence times), carbon, fixed carbon, and the higher heating value of the resulting hydrochar increase.^18^ However, the net energy produced by the overall process is positive.^17^ Therefore, HTC technology simultaneously presents a solution to the waste management of biomass by turning it into a valuable resource for the production of renewable energy.^19^

The research presented in this review first details the different thermochemical processes alongside the possible reaction mechanisms that occur in the reactor. The challenges currently faced in the hydrothermal carbonisation industry alongside the opportunities this technology presents are assessed. More specifically, in order to explore the opportunity of HTC technology, the implementation of a HTC plant capable of processing both the municipal and sewage waste of a small village (Chirnside in Berwickshire) will be assessed (approx. 2250 residents). Collected data on the current and predicted energy demands alongside waste figures and waste disposal techniques will be used to determine if the implementation of a HTC plant can provide a feasible, sustainable source of energy and efficient waste disposal system in Chirnside. More specifically, the feasibility will be determined by calculating the overall energy balance of the process and demand of the village in 2041. In addition, the current developments made in HTC are also explored for Europe, the United Kingdom, America and Asia.

## 2. Thermochemical processes

1In the 21st century, biomass is being converted into a renewable energy source through the global application of numerous industrial technologies and processes. Besides thermal conversion of biomass (combustion), there are currently three main process technologies currently available: bio-chemical, thermo-chemical and physio-chemical. The main reason behind recent interest in bioenergy production is the potentially unlimited supply of biomass available, due to its renewability. Thus, biomass is the only naturally occurring carbon resource that is available in large enough quantities to substitute for the world's primary energy-containing resource (fossil fuels).^20^ Due to this regeneration potential, the carbon-cycle connecting the production and combustion of products is favourable when compared to fossil fuels which are finite.^21^ Therefore, the variety of biomass energy conversion processes present and a viable opportunity to combat both global warming and climate change.

As previously described, thermo-chemical processes use the application of both heat and chemical processing to produce an energy product (biofuel) from biomass. In literature, these processes are often referred to under varying synonymous names, with the reactor conditions terming the specific type. Table 1 summarises the typical process conditions and product distribution of the various thermo-chemical processes.^7–10^ However, it should be noted that the reactor conditions employed will vary depending on the reactor size, feedstock type, product application and technology manufacturer. The significant difference between the thermo-chemical processes identified is the ability of reactors to process feedstocks with a moisture content of 75–90%. In comparison, dry pyrolysis, gasification and torrefaction are unlikely to be driven economically by a moisture content above approximately 50–70%.^7^ Previous to wet pyrolysis, any feedstocks with high moisture contents would require a significant amount of energy to thermally dry the feed before processing. As a result of this unfavourable energy used on intensive pre-treatment of the biomass, the more viable option would be discarding any high moisture biomass feeds. This highlights the importance of hydrothermal carbonisation: a bioenergy process that is capable of processing feedstocks with an elevated moisture content.

**Table 1:** Comparison of thermochemical processes for biomass transformation^7–10^

Process	Temperature (°C)	Residence time	Pressure (bar)	Other conditions	Typical product distribution (weight%)		
					Solid	Liquids	Gases
Gasification	900–1500	10–20 s	1	Limited oxygen supply	10	5	85
				Moisture content 10–20%			
Dry torrefaction (mild pyrolysis)	200–300	1 h	1	No oxygen	80–90	5–10	0–10
				Moisture content <10%			
Slow pyrolysis	350–400	5 min–12 h	1	No oxygen	25–35	20–50	20–50
				Moisture content 15–20%			
Intermediate pyrolysis	350–450	4 min	1	No oxygen	30–40	35–45	20–30
				Moisture content <10%			
Fast pyrolysis	450–550	1–5 s	1	No oxygen	10–25	50–70	10–30
				Moisture content <10%			
Wet	180–250	0.5–8 h	10–40	Moisture content 75–90%	50–80	5–20	2–5
Pyrolysis (HTC)							

### 2.1 Pyrolysis

Derived from the Greek word ‘pyro’ meaning fire and ‘lysis’ meaning ‘to unbind’, this process describes the thermal decomposition of organic material under anaerobic conditions. During a pyrolysis operation, the biomass feed decomposes under high temperatures and pressures to produce an energy dense and carbon rich stream. Pyrolysis can be completed under a variety of process conditions which redefines the process as slow, fast or intermediate, which determines the product yields. For sixty experimental feedstocks, the typical mass yields obtained for biochar, bio-oils and biogases for each slow, intermediate and fast pyrolysis can be found in Table 1.^8^ Feed moisture contents below 10% is recommended for fast pyrolysis to ensure that the rate of temperature rise is not restricted by the evaporation of water.^8^ Whereas slow pyrolysis is more tolerant at a moisture content of 15–20%. However, the main concern associated with slow pyrolysis is the effect of longer residence time on the process energy requirement.^9^ The operating pressure of pyrolysis will strongly influence the yield of biochar produced; experimental data demonstrates that as reactor pressure increases, the product yield increases, independent of feedstock used.^22^ This being said, pyrolysis is often carried out at atmospheric pressure^23^ to minimise energy consumption and associated costs.

### 2.2 Wet pyrolysis (hydrothermal carbonisation)

When pyrolysis is carried out in the presence of subcritical liquid water, the process is redefined as ‘hydrous’ or ‘wet’ pyrolysis, industrially known as Hydrothermal Carbonisation. In comparison to dry pyrolysis, the moisture content of the biomass feed is typically between 75–90%.^7^ This allows HTC to process a variety of non-traditional biomass sources when compared to pyrolysis, such as municipal solid wastes, animal manure and sewage sludge, alongside traditional biomass sources, *e.g.*, wood and grass.^7,24^

In an operational HTC process, the wet biomass is transformed into pellets known as hydrochar through thermal treatment in a pressurised vessel. HTC is distinguished from hydrothermal liquefaction as the hydrochar product is solid, as opposed to a liquid bio-oil.

In comparison to pyrolysis which typically takes place at higher temperatures and atmospheric pressure, HTC reactor conditions are typically within the operating range of 180–250 °C and take place at elevated pressures, typically between 10–40 bar.^10^

In addition to the feedstock type, HTC reactor conditions also affect the property of the resulting hydrochar.^25^ For example, one study conducted HTC of paper sludge over an experimental range of 180–300 °C. The maximum heating value (9.7 MJ kg^−1^) and highest energetic recovery efficiency (90.12%) of the experimental trials was at a temperature of 210 °C.^26^ This implies that final application of the hydrochar as fuel source would be most optimally produced at this temperature. However, this study further found that hydrochar had lower nitrogen and sulphur contents as the reactor temperature was increased.^26^ This implies that a lower reactor temperature would be favoured for hydrochar that is to be applied as a soil conditioner (for a paper sludge feedstock). Furthermore, nitrogen content in hydrochar has been shown to have a significant impact on its specific applications.^27^ By identifying the application of the hydrochar and by analysing the composition of the feedstock, research has shown that the ideal reactor conditions can be determined. In turn, the resulting hydrochar can be optimised for a variety of applications, currently including:

• An independent or co-generative heat and power fuel source.^16,19,28^

• A soil conditioner.^29–31^

• An adsorbent.^13,32,33^

• A supercapacitor electrode material.^34^

• Replacing biomass in co-fired coal plants (preventing fuel segregation in boilers, burnout, inefficiencies and fouling).^35^

## 3. Hydrothermal carbonisation: fundamentals and reaction mechanisms

**Fig. 1:**
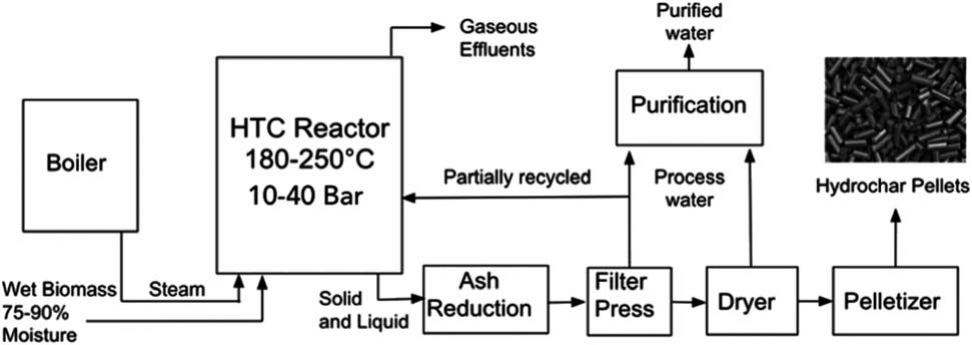
Typical hydrothermal carbonisation process.

Fig. 1 represents the typical process units as defined by the NEWAPP project.^17^ Typically, alongside the elevated temperatures, the closed reactor vessel within a HTC plant is subject to elevated pressures and residence time. The process route, unit dimensions and conditions will vary depending upon technology licencing of the original equipment manufacturers. As shown on Fig. 1, the reactor effluent is subjected to a downstream filter press unit in order to increase the concentration of the carbon content through reduction of the moisture content to near 50 wt%. After that, the process water is removed through filtration and is partially recycled back into the reactor to increase the energy efficiency.^17^ The carbonaceous produced is then subjected to thermal drying, which is an extremely energy intensive process to remove excess moisture before pelletization of the solid hydrochar.

A secondary product amongst the hydrochar pellets is the process water stream that is released from the filter press and thermal drying stages. The process water contains short-chained carboxylic acids and inorganic ions such as potassium and phosphate, both of which are beneficial to plant growth. However, out of 680 organic pollutants tested for in the process water, traces of 13 were detected. These initial results are ‘un-alarming’ for fertiliser applications. However, in the long term, tests on the impact of irrigation with HTC process water in agricultural soils have been recommended.^17^ Alongside the liquid and solid phase products, approximately 5 wt% of the raw materials dry mass will be accounted for the gaseous effluent which consists mainly of carbon dioxide with traces of carbon monoxide and methane.^19^

### 3.1 Structure of biomass

**Fig. 2:**
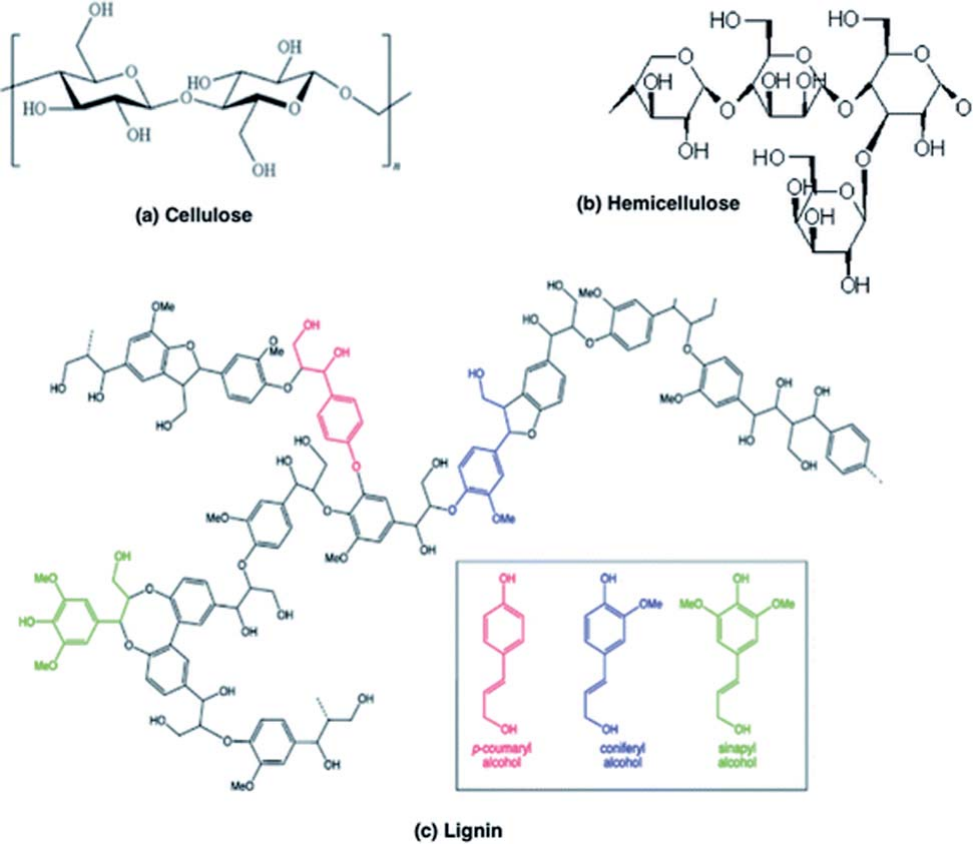
Chemical structures of (a) cellulose, (b) hemicellulose and (c) lignin.

Biomass cannot be defined as a specific reactant due to its high degree of chemical complexity and heterogeneity.^11^ Lignocellulose (plant) biomass consists mainly of three carbohydrate polymers: cellulose, hemicellulose and lignin. Chemical structures of these compounds are shown in Fig. 2. Small quantities of pectin, protein, extractives and ash have also been detected and the composition of all constituents vary among plant species. Cellulose is the main constituent of the plant cell wall and chains of 20–300 monomers group together to form microfibrils. Hemicellulose is the second most abundant polymer, which is not chemically homogeneous and contains branches with short lateral chains of different sugar types (xylan is presented in Fig. 2). Lignin is the third most abundant polymer in nature. Its molecular structure contains cross-linked polymers of phenolic monomers.^36^

### 3.2 Reaction mechanisms

The thermo-chemical conversion of biomass into lignite coal-type hydrochar is a complex reaction network, the exact details of which is unknown.^37^ In order to reach a clearer understanding of the reaction mechanisms that are involved in hydrothermal carbonisation, Infrared (IR) spectroscopy of both biomass feedstock and resulting hydrochar has been utilised.^38^ This appliance promotes the identification of possible reaction mechanisms through the detection of functional groups present in both the feedstocks and product samples; however, it is time-consuming expensive and can require the use of additional chemicals. More recently, hyperspectral imagining has been used to provide a robust and reliable alternative for quantitative determination of polysaccharides in biomass and biomass chars.^39^ Hyperspectral imagining is both fast and non-destructive, and in storing data on-line, decompositions of polysaccharides (and thus resulting qualities of hydrochar) can be predicted from feedstock analysis and comparison.^39^

However and so far, only separate discussions of general reaction mechanisms have been identified to provide useful information about the possibilities of manipulating the reaction. The reaction mechanisms that have been identified for pyrolysis in the presence of subcritical water include hydrolysis, dehydration, decarboxylation, condensation polymerisation and aromatization.^7^ These do not represent consecutive reaction steps but rather form a parallel network of simultaneous reaction paths.^37^

Using cellulose chains as a model biomass substance, the following reaction equations under hydrothermal carbonisation conditions have been deduced from experimental results:

1 (C_6_H_12_O_5_)_*n*_ → *n*C_5.25_H_4_O_0.5_ + 0.75*n*CO_2_ + 3*n*H_2_O

2 Δ*H*_R_ = 1.6 kJ per kg cellulose

Eqn (1) approximates the stoichiometric ratios of reactants to products within an HTC reactor.^6^ However, these approximations have a large margin for error and should be treated with care, as the chemical pathway is not fully defined. Additionally, eqn (1) does not account for the liquid organic reaction by-products that represent an important fraction.^40^ As described by eqn (2), the HTC process is exothermic (negative heat of reaction) for a pure cellulose feed. However, the heat released is highly dependent on feed composition and the reactor conditions. Although eqn (1) and (2) cannot accurately describe the treatment of a biomass stream, these equations can offer an insight of what is to be expected from HTC of lignocellulosic biomass. Thus, the reaction pathways identified for the pyrolysis of the three lignocellulosic carbohydrate polymers can be predicted. This being said experiments by Volpe determined that pure cellulose remained unaltered at temperatures up to 220 °C, yet significantly decomposed at 230 °C to produce recalcitrant aromatic and high energy-dense material.^36^

#### 3.2.1 Hydrolysis

**Fig. 3:**
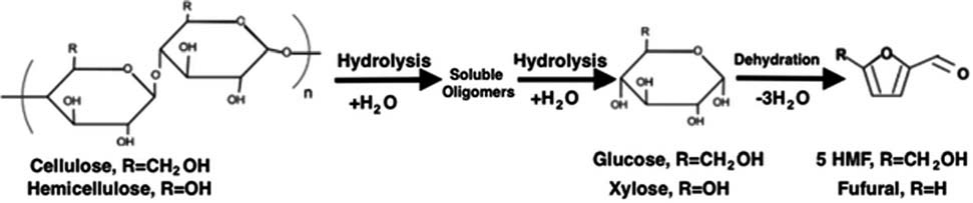
The hydrolysis reaction pathway from cellulose and hemicellulose to glucose and xylose.

Hydrolytic reactions occur on the surface of solid biomass, where water reacts with biomacromolecules by breaking both ester and ether bonds to produce a wide range of products.^4^ Liquid water enters through surface pores and hydrolyses the components, after which the hydrolysed products may proceed to exit out of the same pore. The biomacromolecules (cellulose and hemicellulose chains) are initially hydrolysed into soluble oligomer products. With increased reaction time, the oligomers further hydrolyse into simple monosaccharide or disaccharides.^4^Fig. 3 shows the reaction pathway during further hydrolysis of the oligomers to produce glucose and xylose from cellulose and hemicellulose, respectively. However, the quantity of different fragments formed from the hydrolysis of these polymers is very high and is not limited to the reaction pathway shown in Fig. 3. Alongside this, hydrolysis of lignin is known to produce guaiacol, phenol and catechol.^41^

Through forced convection, hydrolysis can be completed within a few minutes with the rate being determined by the adjusted flowrate, not only the reaction temperature.^42^ Although hydrolysis of lignocellulosic biomass can take place at lower temperatures, significant hydrolysis of cellulose has been found to occur above 220 °C and lignin is most likely realizable at 200 °C due to the high number of ether bonds. And, hemicellulose has been found to readily hydrolyse at around 180 °C.^37^ IR spectroscopy graphs for lignocellulose hydrochar contain no evidence of the presence of hemicellulose, suggesting that hemicellulose is fully hydrolysed at elevated temperatures.^4^ The fragments formed are highly reactive and will quickly undergo condensation reactions to form precipitates.^43^ The rate of hydrolysis during HTC is primarily determined by diffusion and thus limited by transport phenomena within the matrix of the biomass. This may lead to condensation of fragments within the matrix at high temperatures.^44^

#### 3.2.2 Dehydration

Dehydration of biomass is the formation of water molecules *via* the elimination of branched hydroxyl (–OH) groups, also known as dihydroxylation. This reaction produces hydrochar with a lower O/C and H/C ratio when compared to the feed and replicates the ratios present in natural coal. However, the complete chemical structure varies significantly between these two fuels. The ratio of O/C and H/C bonds is inversely dependant on the temperature, and more significantly so for O/C bonds.^45^

The products resulting from the hydrolysis of cellulose and hemicellulose are dehydrated to form 5-hydroxymethylfurfural (HMF) and furfural, respectively, as shown in Fig. 3. Dehydration of water during the cleavage of both phenolic monomers and hydroxyl functional groups may occur during HTC at temperatures above 150 °C and 200 °C, respectively. The dehydration of catechol, formed from the hydrolysis of lignin, may also occur.^37^

Dehydration (and decarboxylation) occurs in the HTC process as both residence time and temperature increase.^46^ Alongside the manipulation of these conditions for improved efficiency, additives that promote the rate of reaction can be combined into the feedstock to support and accelerate this reaction mechanism. For example, alkaline conditions give the highest reaction rates for hydrolysis whereas further degradation reactions of simple mono- or disaccharides are highly enhanced under acidic conditions^43^ using most commonly mineral acids such as sulphuric and hydrochloric acids.^47^

#### 3.2.3 Decarboxylation

IR spectroscopy graphs for hydrochar demonstrate no peak detection around wavenumber 1725 cm^−1^.^4^ This suggests complete carbonyl (–C=O) and carboxyl (–COOH) degradation and can be associated to the formation of carbon monoxide (CO) and carbon dioxide (CO_2_), respectively.^4^ Carbonyl and carboxyl degradation occur rapidly at temperatures above 150 °C to produce minor concentrations of the gases mentioned prevsiouly.^48^ The carbonyl functional group is presented on both 5-HMF and furfural molecules (Fig. 3), and the likely source of the carboxyl functional group is the formation of both formic acid and levulinic acid (the hydrolysis products of furfural and 5-HMF, respectively).^49^ Research publications confirmed the major portion of gaseous products from HTC to be CO_2_.^50,51^ However, more CO_2_ is produced than can be explained by the elimination of carboxyl groups alone.^45^ This suggests that other mechanisms are involved in the process; likely carboxyl group sources have been identified as products from condensation reactions^45^ and the cleavage of intermolecular bonds.^52^ Alongside this, experimental evaluations of the carbon monoxide produced during HTC is insufficient to account for the loss of all carbonyl groups. This suggests that carbon dioxide may be formed from their degradation.^48^ It should be noted that dehydration and decarboxylation occur simultaneously with significant decarboxylation appearing after a significant amount of water has been formed.^6^

#### 3.2.4 Condensation polymerisation

Some of the fragments formed from the degradation of biomacromolecules are highly reactive (*e.g.*, anhydroglucose, 5-HMF, aldehydes, lignin fragments).^4^ The unsaturated carboxyl and hydroxyl groups polymerise easily^53^ and this leads to the formation of a water molecule (condensation) and ether bonds (–COC–).^37^ Condensation polymerisation is most likely governed by step-growth polymerisation which is enhanced at higher temperatures and reaction time. Highly reactive lignin fragments have been reported to polymerise in several minutes at 300 °C, whereas at room temperature polymerization can continue for months.^54^ The rate of polymerisation during HTC is similarly temperature dependant; ether bond in IR graphs of HTC hydrochar becomes more pronounced as the reactor temperature is increased.^4^ Thus, the formation of the lignite-structure of hydrochar is mainly characterised by condensation polymerisation.^42^ However, the knowledge about the detailed polymerization sequences during the course of hydrothermal carbonisation is essentially missing.^37^

#### 3.2.5 Aromatization

Lignin is naturally composed of many stable aromatic rings, as shown in Fig. 3. These aromatic structures exhibit high stability under hydrothermal carbonisation conditions and are considered to be the basic building block of the resulting hydrochar.^37^ The IR spectra graph for lignocellulose's hydrochar product have shown that the peak corresponding to aromatics (1694 cm^−1^) is enhanced when compared to that of the raw feedstock.^4^ Alongside this, experiments have shown that increasing the reactor residence time and/or reactor temperature leads to an increased percentage of lignin, which is more than that of raw feed. To quantify, one experiment measured a percentage mass of lignin in the raw biomass feed as 7%. Operating the pilot HTC reactor at 200 °C for 1 and 6 hours found a percentage mass for pseudo lignin of 25.1 and 38.3%, respectively. Whereas operating at 250 °C for the same residence time found 44.4% and 58.3%, respectively.^15^ Therefore, conclusions have been drawn that the lignin-like substances that are formed (pseudo lignin) during HTC conditions result from the aromatization of cellulose and hemicellulose, despite there being linear carbohydrate polymer chains.^4,55^ The structure of hydrochar is concluded to be in agreement with natural coal, as the cross-linking condensation of aromatic rings makes up its major constituent.^56^ As shown, aromatization and the concentration of pseudo-lignin in hydrochar is significantly dependant on temperature.^53^ In addition, it has been determined that the formation of aromatic structures have been enhanced by alkaline conditions.^57^

#### 3.2.6 Other mechanisms

Other minor mechanisms that may occur under the hydrothermal carbonisation of biomass include:

• Demethylation.^58^

• Pyrolytic reactions.^37,59–61^

• Fischer–Tropsch reactions.^62^

• Transformation reactions.^46,63^

• Secondary char formation.^18^

The catechol-structure of the coal is thought to be explained by the demethylation of phenol.^58^ This is commonly the replacement of a methyl group (–CH_3_) with a hydrogen atom. This mechanism is supported by the production of minor amounts of methane that has been observed over several experiments.^37^

Alongside this, pyrolytic reactions have been reported to be competing reactions when under hydrothermal conditions.^57^ In general, they might become more significant above 200 °C,^58^ though typical products from pyrolysis have not been reported to be formed in significant amounts during hydrothermal carbonisation.^37^ They are thought to occur due to fragments of the feedstock that have not come into contact with water due to being trapped within the biomass matrix by the precipitation of condensed fragments.^60^

Fischer–Tropsch reactions have also been observed under hydrothermal conditions.^62^ A high amount of CO_2_ is formed during hydrothermal carbonisation and the Fisher-Tropsch reactions may play a role in the production of this gas that has not been investigated in detail so far.

Transformation reactions within the lignin may occur when the hydrolysis and subsequent condensation (polymerisation) cannot take place. This is mainly for stable compounds with a crystalline structure and oligomer fragments as these do not hydrolyse.^63^ However, given the high rate of fragmentation by degradation due to hydrolysis above 180 °C, it appears unlikely that transformation reactions play a key role under hydrothermal conditions.^37^

In addition, solid secondary chars have been determined to form from the liquid depolymerized cellulose anhydro-oligomers formed in pyrolysis.^64^ Similary, Lucian *et al.* writes that the formation of hydrochars from the hydrothermal carbonisation of the organic fraction of municipal solid waste forms a reactive secondary chars on the surface of the primary hydrochar, suggested from the thermal stability and reactivity of the intermediate hydrochars.^18^ Extracting and experimenting, the HHV of the secondary chars in this study was found to be significantly higher than those of the primary char that was formed.^18^

## 4. Research, development and application

The support for the research, development and application of HTC technology is based on the promising positive contribution the technology is expected to have, within both fields of renewable energy production and waste biomass disposal. There are an estimated 200 companies and organisations distributed worldwide that are currently involved in the research, development and application of HTC technology. In 2013, 150 patents concerning the hydrothermal carbonisation process were processed, of which 39% were from cross-country collaborations, China (27%), America (14%), Germany (10%) and Others (10%).^17^

### 4.1 Europe

#### 4.1.1 Incentive in Europe

Before the development of HTC, wet biomass feedstock was sent to landfill, directly incinerated or transformed by the alternative thermochemical methods outlined in Section 2. However, new approaches to waste management are proceeding in compliance to the requirements of the Landfill Directive (1999/31/EC). This directive is composed by the European Commission who proposes legislations for the EU member countries. In 2014, the European Commission outlined landfilling as the least preferable option of waste disposal.^65^ Alternatively, direct incineration of biomass with a high moisture content should be avoided due to the low energy efficiency that results from the large quantity of energy required for the evaporation of water. Alongside diversion of waste from landfills, the European Commission have set a ‘binding’ target to achieve 20% of the EU's final energy consumption to be from renewable sources by 2020. The 2030 target was originally at 27%. However, recent revision of the Renewable Energy Directive increased the target to 32% as the EU aims to be a global leader in renewable energy production.^66^

#### 4.1.2 Support for HTC research and development in Europe

The European Union is actively involved in the support and funding of innovation within HTC research, development and its multi-market application. Programmes such as Horizon 2020 and the EUs 7^th^ Framework were established by the European Union with the aim to tackle the biggest challenges within transportation and energy sectors that currently face modern society.^67^

Alongside this, the European Biomass Industry Association has coordinated projects such as the ‘new technological applications for wet biomass waste stream products’,^17^ which received a contribution of €1.76 million from the EU. One of the main targets of this research was to produce a draft of quality standards for hydrochar that is to be used as a solid fuel and as a soil conditioner (in cooperation with the Organisation for Standardization (ISO)).^17^ The formation of these standards was deemed necessary in order to prove the viability of hydrochar in commercial applications. Establishing standards allows hydrochar manufacturers to receive certification based upon the quality of their product and in turn market growth is stimulated as the product and technology is trusted by investors/clients.

In preparation of these standards, Project NEWAPP identified the following 5 substrate streams as feedstock which were then tested and analysed from potential suppliers to assess its suitability to the HTC process:^17^

• Sewage Sludge – from wastewater treatment plants.

• Digestate – from anaerobic digestion plants.

• Green waste – vegetables, pruning *etc.*

• Household food waste.

• Organic fraction of municipal solid waste.

The standards established from the experimental testing of these streams are taken as the basis of calculation for the energy balance produced in Section 6. Alongside the many experimental trials performed, project NEWAPP conducted a comparative economic analysis of hydrochar to other fuel sources and a comparative economic analysis of HTC to other waste disposal methods. The results are considered when discussing the opportunity and challenges HTC presents to the UK in Sections 7 and 8, respectively. In addition, the impact assessment of the comparative environmental life cycle assessment study concluded that application of hydrochar as a fuel source is more suitable than application as a soil conditioner.^17^

#### 4.1.3 Research, development and application in Europe

Europe is leading the way through commercial and industrial application of HTC technology. Recent studies have investigated the hydrothermal carbonisation of olive mill waste, resulting from the production of olive oil, which has demonstrated very positive results; high heating values of 32.3 MJ kg^−1^, alongside improved fouling and slagging properties than the direct combustion of olive mill waste.^14^

European researchers have been collaborating internationally to assess the viability of implementation in alternative markets. For example, researchers from Berlin have investigated the feasibility of the hydrothermal carbonisation of empty fruit bunches (EFB) that result from the production of palm oil in Indonesia and Malaysia.^14^ Similarly, researchers from Switzerland have worked with academics in Thailand to characterise the hydrochar produced from the HTC of bamboo.^18^

Noticeable companies developing a HTC process in Europe include Ingelia (Spain), C-Green (Sweden), HTCycle (Germany), SunCoal (Germany) and AVA-CO_2_ based in Switzerland with subsidiaries in Germany.

Ingelia is one of a handful of recent companies founded with the purpose of providing the technology for hydrothermal carbonisation. This is the first industrial HTC plant worldwide capable of carbonizing wet biomass in a continuous process.^68^ The HTC process design produced by Ingelia is modular, which allows scalability for a client's specific needs and future plant expansion.

In mid-2018, C-Green €2.2 developed a full-scale HTC plant in Heinola, Finland, capable of processing 25 000 tonnes of residual biomass per year that is currently produced by StoraEnso's corrugated board mill.^69^

HTCycle and SunCoal are based in Germany where they too are collaborating with partners and clients to commercialise their patented HTC technology. Alongside offering services for HTC technology, SunCoal have developed an entrained-flow gasifier for the production of syngas from hydrochar.^70^

In 2010, AVA-CO_2_ had claim to the world's largest HTC demonstration plant based in Karlsruhe, Germany, with a production capacity of 1000 tonnes of hydrochar per year.^71^ After which, AVA-CO_2_ constructed and commenced operation in an industrial-sized multi-batch HTC plant in 2012, with production capacity of 8000 tonnes of hydrochar per year.^72^

### 4.2 The United Kingdom

#### 4.2.1 Research, development and application in the United Kingdom

The contributions to research within the field of HTC technology continue to increase from academics based at universities across the UK. Noticeable contributions come from the University of Edinburgh, Queen Mary University of London, the University of Nottingham and Loughborough University.

Uniquely, academics from Loughborough University have progressed beyond experimental research as they have developed a small-scale HTC toilet system.^73^

Noticeable companies in the UK include clean-tech start-ups such as Antaco and Valmet. Due to the commercial potential of their patented process, Antaco completed construction on its pilot plant in 2014 making it the first HTC plant in the UK (not of commercial scale).^74^

Valmet and previously discussed German-based company SunCoal have joined forces with the focus on the HTC processing of sludge derived from the paper and pulp industry for.

### 4.3 Research and development in Asia

As mentioned previously, besides those from multiple-country-collaborations, the majority of applications for HTC patents, come from China (27%). Research conducted by Zhou *et al.* (2018) has shown that the weight percentage of food waste in municipal solid waste (MSW) in cities throughout China ranges from 30–60%. This range is larger than that in the following individual countries: USA, Germany, England, Japan and Singapore.^75^

Application of HTC in China has already begun; an HTC plant that processes 14 000 tonnes of sewage sludge per year is operated in Jining.^76^

However, Asia has been exploring the HTC processing of alternative wastes compared to the UK, such as waste textiles (China)^77^ coconut fibre and eucalypts leaves (Singapore)^43^ and seaweed (Japan and Indonesia)^78^ due to the high production potential of both biomass sources there.

## 5. Current methods for biodegradable municipal waste, sewage waste and final treatment waste

As discussed, research, development and application of hydrothermal carbonisation is continuing to grow. In the 21st century, HTC technology companies worldwide are being founded and industrial-sized plants have commenced operation. Alongside this, commercial plants within a multitude of markets have been established through collaboration with the companies who have patented their technology. This section provides the estimate data of biodegradable municipal waste (BMW) and sewage waste produced in the UK alongside current waste disposal methods used. From this, an in-site into the potential supply of BMW and sewage waste biomass for HTC processing in the UK is assessed.

### 5.1 Biodegradable municipal waste

The enforcement of the environmental policies set by the EC directives is covered by the Scottish Environmental Protection Agency (SEPA) in Scotland under the Department for Environmental Food & Rural Affairs (DEFRA). DEFRA, in compliance to the EC directives are required to release yearly statistics of relevant data to prove compliance with the established standards and targets. Recent available data for the UKs Statistics on Waste is the 2016 report produced by the Government Statistical Service.^79^ The key points of relevance in this report are defined as follows:

• UK Biodegradable Municipal Waste (BMW) sent to landfill has continued to reduce and in 2015 was 7.7 million tonnes. This represents 22 percent of the 1995 baseline value. There is an EU target to restrict BMW landfilled to 35 per cent of the 1995 baseline by 2020.

• Of the 209.0 million tonnes of all waste that entered final treatment in the UK in 2014, 44.5% was recovered (including recycling and energy recovery). The proportion that went to landfill was 23.1 percent.

The Scottish Government launched Scotland's first zero-waste policy on the 9th of June 2010. This plan envisions a zero-waste society in which all waste is acknowledged as a viable resource.^80^ From this, waste produced by Scotland's residents and businesses is to be minimised and valuable resources are not to be disposed *via* landfill sites. This initiative action defines that new measures are to be taken by local councils. These measures include:

The banning of specific waste types from landfills in order to capture the value these resources hold.

Restrictions on the energy input to municipal waste facilities (incineration) to encourage waste prevention, reuse and recycling.

Application of HTC technology could be beneficial to the achievement of these measures. However, to date, there has been no investigation by the Scottish Government into the employment of HTC technology in the country. The findings presented in this report will be the first.

#### 5.1.1 Biodegradable municipal waste sent to landfill

Biodegradable Municipal Waste (BMW) is defined in the Landfill Directive (1999/31/EC) as household waste that is capable of undergoing anaerobic or aerobic decomposition. Here, biodegradable fractions are noted to include paper, card, green waste, food waste, miscellaneous combustibles and fines. The aim of the Landfill Directive is to prevent or reduce, as far as possible, the negative impacts on groundwater, soil, air and human health that are associated with the landfilling of waste. This is achieved through the stringent technical requirements established for the UK to achieve. One of which is to reduce the amount of BMW sent to landfill as uncontrolled decomposition of BMW leads to the production of landfill gases.^81^ This gas mainly consists of carbon dioxide and methane, both of which are greenhouse gases. And, methane gas is 20 times more potent than carbon dioxide in its impact.^82^

The 2010 target defined in the Landfill Directive states that UK should aim to reduce the tonnage of BMW sent to Landfill to 35% of the baseline by 2020. Table 2 (data from Department for Environment, Food and Rural Affairs)^79^ shows the percentage of the 1995 target baseline of BMW sent to Landfill for each country in the UK from 2010 to 2015. It should be noted that biodegradable municipal waste for each country (bar Northern Ireland) represents approximately half of the overall municipal waste sent to landfills in the UK. Table 2 demonstrates that the UK has achieved and even improved upon the target established in 2010 set to control BMW sent to landfill; the overall percentage in 2015 has been reduced to 22% whereas the target was to reach 35% of the 1995 baseline by 2020. This demonstrates that the UK has significantly reduced the amount of BMW produced and/or took affirmative action for BMW diversion from landfills. This being said, 7682 kTnes of BMW that could have been treated through HTC was sent to landfill in 2015.

**Table 2:** BMW sent to Landfill in the UK and country split and the % representation in comparison to the 1995 baseline^79^

Year	Mass of BMW sent to landfill per year (kTonnes per year)					Percentage value to baseline (%)				
	UK	England	NI	Scotland	Wales	UK	England	NI	Scotland	Wales
1995	35 688	29 030	1225	3595	1837	—	—	—	—	—
2010	12 982	10 339	558	1484	600	36	36	46	41	33
2011	11 719	9360	464	1358	538	33	32	38	38	29
2012	10 337	8129	394	1292	522	29	28	32	36	28
2013	9326	7347	299	1183	497	26	25	24	33	27
2014	8711	6843	322	1122	424	24	24	26	31	23
2015	7682	5980	307	1084	311	22	21	25	30	17

**Fig. 4:**
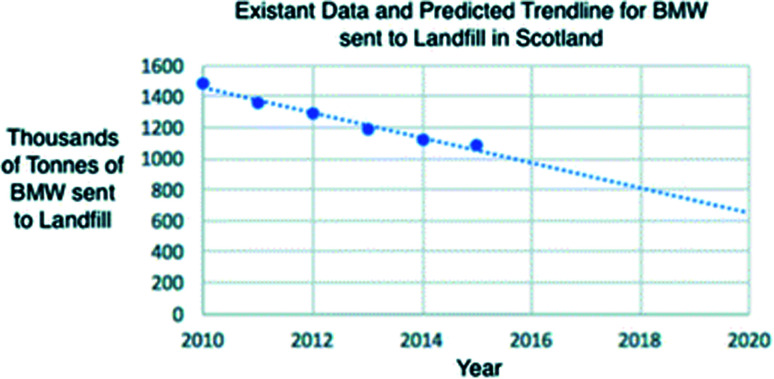
A graph to show the current data and predicted trend line for the BMW to Landfill in Scotland.^80^

Although surpassing the 35% target established by the EU by 5%, Scotland is the lowest performing country in the UK at reducing the amount of BMW sent to landfill. The linear trend shown in Fig. 4 indicates a future prediction of BMW sent to landfill in Scotland based on previous data.^79^ By 2020 the quantity of BMW sent to landfill is predicted to be approximately 6 million tonnes if efforts for its reduction are continued.

#### 5.1.2 Biodegradable municipal waste sent to incineration

The largest reduction in BMW sent to landfill was in Wales which saw a 6% drop in the years 2014 to 2015. The UK Statistics on Waste identified this considerable reduction to be attributed to an energy-from-waste plant becoming fully operational in Cardiff. The type of energy-from-waste plant was found to be an incineration plant owned by Viridor Ltd. Proven to aid in the reduction of BMW sent to landfill, the incineration of biomass is a source of energy. More specifically, Viridor's thermo-chemical incineration plant is capable of generating 30 MW of electricity for the national grid (∼50 000 households) and handles 350 000 tonnes of residual waste per year. The carbon footprint calculated in association with Viridor's incineration plant is lower than that produced by landfill. It is also lower than the carbon footprint produced from the conventional fossil-based electricity and heat generation.^83^

DEFRA recognises that a Municipal Solid Waste (MSW) stream will contain both carbon-based (biomass derived material) and fossil-fuel based products. Incineration of biomass in MSW is a renewable source of energy, as this biogenic portion is ‘capable of being replenished, not depleted by its utilization’ (OED). However, incineration of fossil-fuel based products is not a renewable source of energy as the emissions released from their combustion contribute to the greenhouse effect and global warming.^84^ In turn, defining the overall process of incineration as ‘renewable’ is incorrect. Alongside greenhouse gas emissions, incineration of waste has the potential to release various harmful and carcinogenic emissions including acid gases, nitrogen oxide, heavy metals (lead), particulates, dioxins and furans.^70^ Thus, some air-pollution control techniques are implemented in plant designs (NO_*x*_ control, acid gas scrubber, continuous emission monitors, *etc.*). However, emissions from incineration is inevitable. Alongside this, data required for necessary health-effect assessments, specifically data on the most harmful emissions (dioxins, furans, heavy metals and particulates) are not readily available from operating plants.^85^ Therefore, the escape of these carcinogenic compounds cannot be overlooked when considering incineration of waste alongside sustainable future development. From this, it can be concluded that incineration of biomass is renewable, while current incineration methods are not sustainable due to the combined processing of biomass with fossil-fuel based products. Therefore, when comparing incineration with hydrothermal carbonisation of waste, HTC presents a more sustainable energy-from-waste process as there is no association with the release of harmful/carcinogenic emissions.

### 5.2 Sewage treatment

Urban waste water, commonly referred to as sewage, is composed of domestic waste water from baths, sinks, washing machines and toilets, alongside industrial waste and rainwater machines and toilets, alongside industrial waste and rainwater collected from drains.^72^ The sewer system in the UK collects over 11 billion litres a day which is equivalent to 4400 Olympic swimming pools. This water is treated at one of the 9000 sewage treatment plants in the UK before being discharged into inland waters. Through extraction of organic substrates from wastewater, the discharged water will have a concentration of biological oxygen demand (BOD) deemed safe by DEFRA standards for aquatic life to survive.^86^

There are four types of treatment that waste water can be subjected to:

• Preliminary treatment – removal of grit, gravel and larger solids.

• Primary treatment – Settling out of any solid matter (removes ∼60% of solids and ∼35% of BOD).

• Secondary treatment – the use of digestate bacteria to breakdown organic substances (removes ∼85% of BOD and solids).

• Tertiary treatment – disinfecting/denitrification of the treated effluent (to protect sensitive water environments from eutrophication).

Typically, sewage waters contain less than 0.1% of solid matter. And once separated in the primary treatment, the resulting ‘sludge’ contains organic matter, dead bacteria from the treatment process and any particulates.^87^ It is this biomass-rich sludge that can be processed in a HTC reactor. Historically, a quarter of the sludge was dumped at sea or discharged to surface waters. However, the EC Directive required the cessation of these practices in 1999. Increasingly, sewage sludge is being processed under anaerobic digestion in which bacteria consumes some of the organic matter in the sludge to produce biogas, a renewable energy source which can be used in combined heat and power plants for electricity generation.^86^

**Fig. 5:**
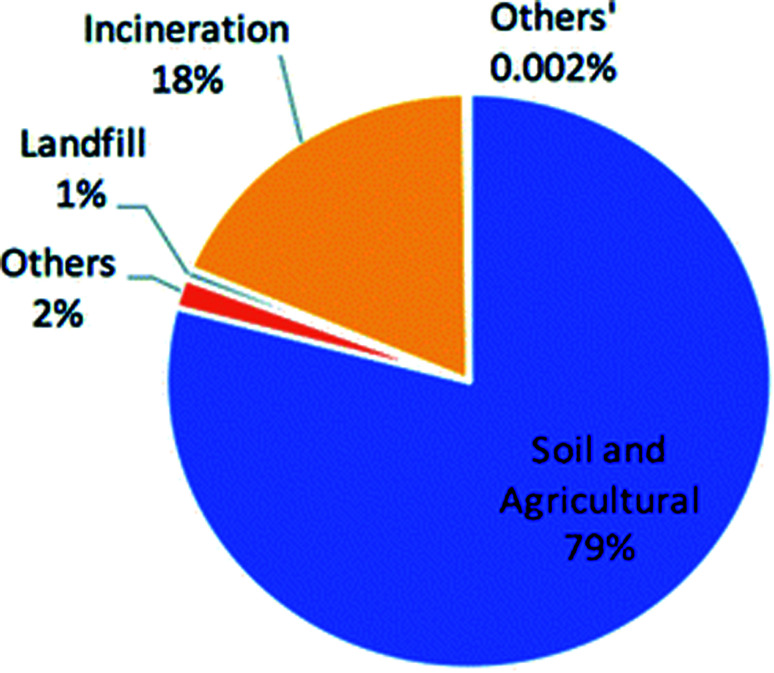
Sewage sludge disposal techniques in the UK (2010).^79^

Fig. 5 displays the percentage split of sewage sludge across its current disposal methods including landfill, incineration and the reusable disposal techniques that include soil and agricultural applications and others.^79^ Clearly, the majority of the UKs sewage sludge is currently reused as a soil enhancer to fertilise agricultural lands, which is considered to be the ‘environmentally favoured option’ by DEFRA. Due to the direct application of sewage sludge as a soil enhancer, processing this waste through a HTC reactor to produce hydrochar pellets for soil enhancement applications would therefore be an inappropriate use of energy.

In 2010, incineration accounted for 18.4% of the disposal of the UKs sewage sludge.^88^ This was the only energy generating application of sewage sludge currently in the UK. As previously discussed, incineration of wet biomass is energetically inefficient as the water content in the sludge requires a large energy input (latent heat) for evaporation. This demonstrates both the advantage and opportunity application of HTC technologies can have in the UK due to the ability to process high moisture feeds. Therefore, HTC of sewage sludge compared to its direct incineration should be considered for sustainable future development in the UK, as energy consumption can be reduced.

### 5.3 Final treatment of waste

**Fig. 6:**
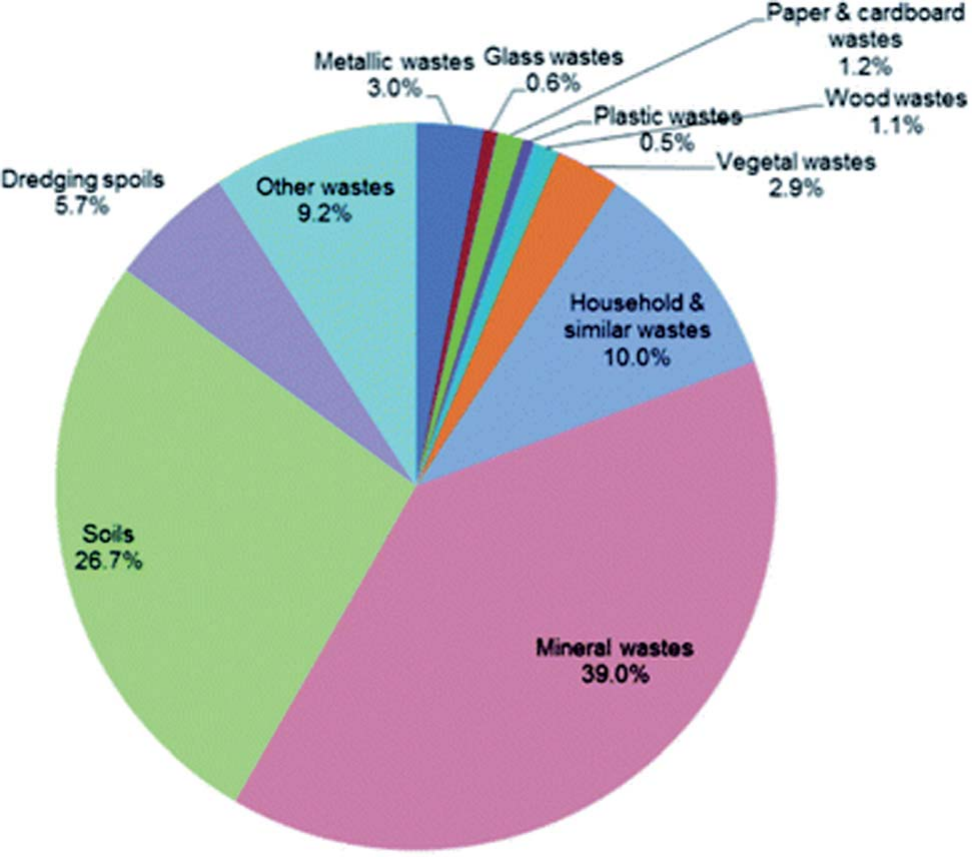
Percentage split of the UKs waste generation by waste material (2014).^79^

DEFRA currently identifies eleven categories of waste in the UK and six final treatment methods. The percentage split of the waste generated in the UK (2014) over the eleven subcategories of waste materials are displayed on Fig. 6.^79^ The waste generated from mining-and-quarrying extractions (mineral wastes) and from soils accounts for two thirds of the overall waste generated in the UK. Considering the other categories presented on Fig. 4, Hydrothermal Carbonisation is capable of processing household, paper & cardboard, wood and vegetal wastes which accounts for a total of 6.2% of the UKs overall waste; a total tonnage of 29.7 million. It should be noted that households and similar wastes are not solely generated by households and this figure does not account for sewage waste.

**Fig. 7:**
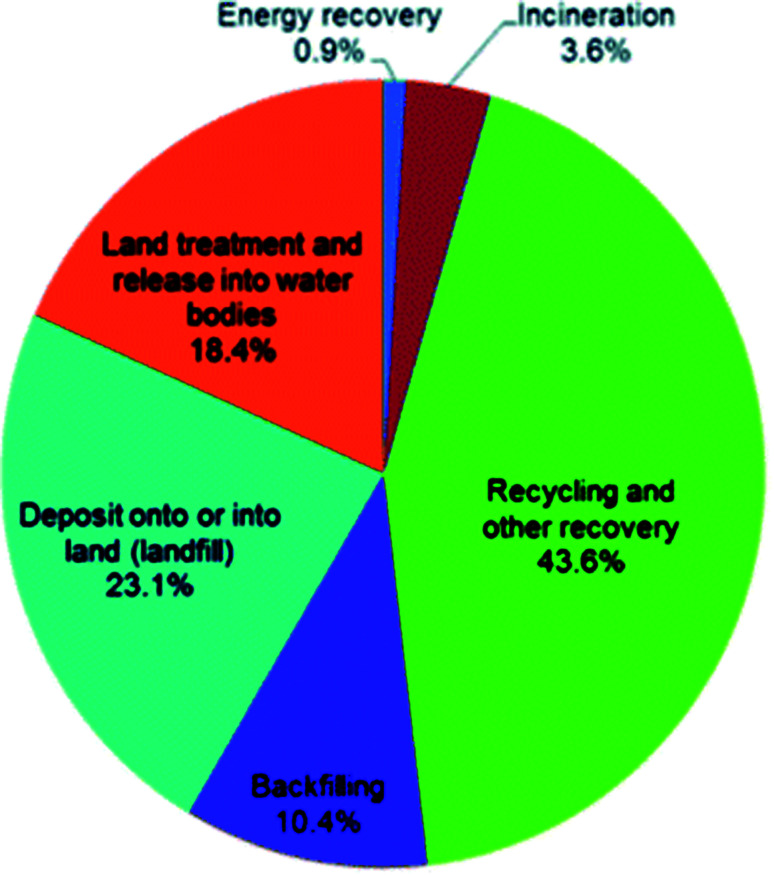
Percentage split of the final treatment method optimised for the overall waste generated in the UK in 2014.^86^

The six final treatment methods, in order of majority percentage are defined by DEFRA as recycling and other recovery, landfill, land treatment and release into water bodies, backfilling and incineration and energy recovery. Fig. 7 is a visual representation of the percentage split amongst these final treatment methods.^86^ Although the majority of waste in the UK is recycled, there is clearly little investment into energy recovery and incineration processes (total 4.5% in 2014).

## 6. HTC application in chirnside, Scotland

### 6.1 Project brief

In order to make progress for sustainable future development, we rely on the continual research and commitment from dedicated scholars into alternative energy production and waste disposal processes. The following section will assess the feasibility of operating a HTC plant in Chirnside, a small village in Scotland. This plant will be capable of processing the biodegradable municipal waste (BMW) and sewage waste produced by the estimated village population in 2041. Calculation of the associated energy balance around the HTC plant design, alongside comparisons to the current waste disposal methods employed in Chirnside, will determine the feasibility of implementing a HTC plant in this village.

### 6.2 Chirnside: research

#### 6.2.1 Population

**Fig. 8:**
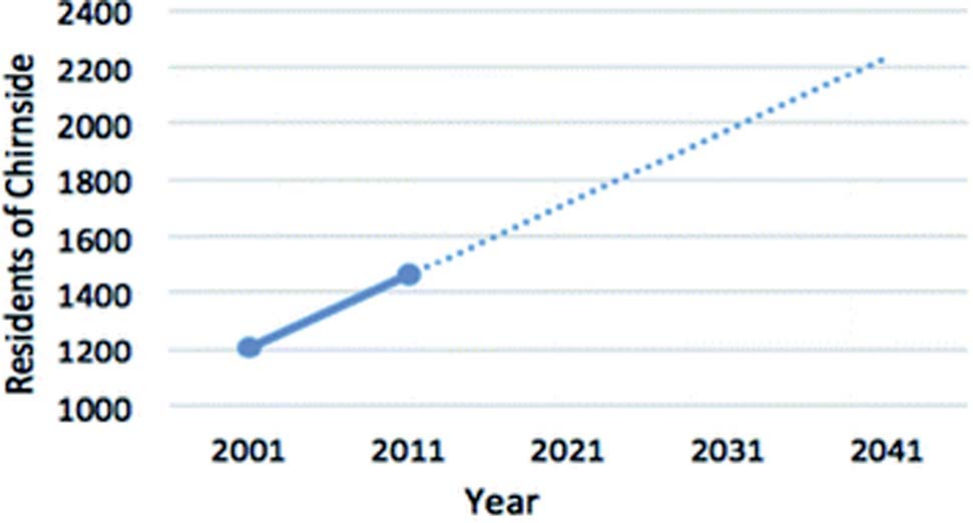
Predicted population growth in Chirnside.^80^

Chirnside is a small village located in the Scottish Borders and is operated by the Scottish Borders Council. The most recent population count in 2011 identified 1460 residents.^89^ However, the difference in this figure and the resident numbers recorded in the 2001 consensus and the approval of recent housing developments in Chirnside by the Scottish Borders Council (46 houses approved in 2010;^90^ 25 houses approved in 2017;^91^ 57 houses applied for planning permission in 2018 (ref. 92)) demonstrates the need to estimate an appropriate population growth. Fig. 8 represents the linear relationship between the population of Chirnside and the years.

In order to appropriately size a HTC module for Chirnside that is capable of processing the towns waste, the population at the time of decommissioning must be estimated. Assuming construction of the plant is completed in 2021, and assuming a 20 year life expectancy of the reactor unit,^93^ the population of Chirnside is estimated to be 2250 in 2041. Various factors can influence this estimate such as fertility, mortality, housing developments and house prices, which in turn will impact the estimated processing rate and plant size. However, the population estimate is appropriate as if the plant is not at capacity, it is assumed that waste from neighbouring municipalities or the agricultural sector can be processed here.

#### 6.2.2 Chirnside: biodegradable municipal waste management

##### 6.2.2.1 Current biodegradable municipal waste disposal techniques

Chirnside is classified as a ‘rural’ area by the Scottish Borders Council and therefore their municipal waste, both food and garden, is not collected for disposal. The Scottish Borders Council suggests each homeowner composts these wastes, and they provide a free home composter to households which do not receive garden and food waste collection. General waste and recycling are collected once every two weeks and recycling centres are located at larger communities in the Scottish Borders, such as Peebles or Eyemouth.^94^ The percentage of Chirnside's residents who use a home compost and those who put their food waste in the general waste bin is unclear. However, for those who do not home compost, it is estimated that more than 30% of the waste in an average bin will be food. This presents an inefficient use of potential resources in Chirnside, unlike several villages in the Scottish borders.

(Galashiels, Hawick, Jedburgh, Peebles, Selkirk and Tweedbank) where the food waste is collected separately for recycling.^92^ This demonstrates that HTC of waste could provide a more sustainable solution to the disposal of food waste in Chirnside, diverting food waste that is currently sent to landfill or composted by transforming the waste into renewable energy.

##### 6.2.2.2 Estimating the food waste in chirnside (changed)

Data on the municipal waste produced in Chirnside is minimal. In order to approximate a figure for the domestic food waste produced in Chirnside, the estimated household food and drink waste figure for Scotland in 2012 is used^95^ together with the population of Scotland in the same statistical year^96^ to calculate an average food waste figure per person per year in Scotland. This was calculated as 118.73 kg per person per year of domestic food waste in Scotland (rounded to 2 dp††Where a number is rounded to 2 decimal places the rounded number is carried forward in all calculations.), allowing for the estimated food waste in Chirnside in 2041 to be estimated as 267.14 t per year. See Table 3 for a summary of these quantifications. As the data for the household food waste produced in Scotland and Population count are in reference to 2012, it is assumed that the amount of food waste produced per person in Scotland is constant for the proceeding calculations. In addition, for further calculations, it is assumed that the 267.14 t per year of domestic food waste produced in Chirnside is all solid food waste and does not include liquid drinks.

**Table 3:** Estimated data (*) to calculate the current municipal food waste in Chirnside in 2041

Term	Value	Unit
Household food and drink waste in Scotland (2012)^95^	630 000	t per year
Population of Scotland in 2012 (ref. 96)	5 306 000	Persons
Average domestic food waste produced per person per year in Scotland (2012)*	118.73	kg per person per year
Estimated population of Chirnside in 2041*	2250	Persons
Estimated domestic food waste in Chirnside in 2041*	267.14	t per year
Estimated average moisture content of domestic food waste^97^	72.95	%
Dry basis mass of domestic food waste produced in Chirnside 2041*	72.26	t per year
Water content of domestic food waste produced in Chirnside 2041*	194.88	t per year

In order to calculate the solid mass of hydrochar produced during Hydrothermal Carbonisation, and to ensure adequate plant design, it is important to understand the dry basis and moisture contents in the waste streams to be processed. As the moisture contents of food waste can vary significantly depending on the type of organic matter, an average moisture content has been assumed; drawing on the literature this is assumed to be 72.95%.^97^ This implies that the domestic food waste produced in Chirnside has a dry basis (no moisture content) mass of 72.26 t per year (2dp) and water content of 194.88 t per year (2dp).

#### 6.2.3 Chirnside: sewage waste management

##### 6.2.3.1 Current sewage waste disposal techniques

Chirnside's wastewater is currently treated by Scottish Water at their small wastewater treatment site, located in Chirnside. This wastewater treatment (WWT) plant is licensed to discharge 318 m^3^ of treated final effluent per day to a standard of less than 25 mg L^−1^ BOD and 10 mg L^−1^ of suspended solids.^98^

The Scottish Borders Council stated in the 2016 Chirnside Local Development Plan that ‘Chirnside has a limited capacity in respect to the waste water treatment works located here and contributions by developers may be required where upgrades are necessary’.^94^ From this, it can be interpreted that necessary investments could be assigned to the potential upgrading and expansion of the current waste water treatment site, and/or contributed to the construction of a HTC plant in Chirnside for sewage sludge processing.

##### 6.2.3.2 Mass of sewage waste produced in Chirnside

It has been estimated that the average person produces a median faecal sludge (dry basis faecal matter plus water content) mass of 128 g per day.^99^ From this data, the amount of “wet” faecal sludge produced by the 2250 residents of Chirnside in 2041 is estimated as 105.12 t per year.

As previously mentioned, understanding the dry basis and moisture contents of the waste streams being processed through HTC is required for plant design and mass balance calculations. The study by Danso-Boateng *et al.*^100^ investigated the hydrothermal carbonisation of faecal sludge where the moisture content of the faecal sludge was determined to be 8.17%.^100^ Using this, the annual mass of water in the faecal sludge for Chirnside's residents is estimated as 8.59 t per year (2 dp) and the annual dry basis faecal matter Chirnside is 96.53 t per year (2 dp).

This being said, it is important to consider that faecal sludge is transported to processing plants *via* the sewerage system through a water medium. The water on its own is referred to as sewage water and when mixed with the faecal sludge it is referred to as primary sewage sludge. As the hydrothermal carbonisation of biomass can take place with moisture contents as high as 75–90% (ref. 101) it is assumed that the total mass of dry biomass (dry faecal matter plus dry food water matter) to water ratio (DB/W) at the Chirnside plant is 0.145; this implies that 14.5% of the reactor mass is solid and the remaining 86.5% is water (including moisture contents of materials). In knowing this ratio, the mass of water in the faecal sludge and the water contents in the food waste, the total mass of sewage water in the primary sewage sludge can be calculated. To qualitatively explain, the total mass of dry biomass is calculated as 168.79 t per year, and in order to achieve the DB/W ratio of 0.145, the additional sewage water permitted to enter the plant in addition to the water content in both food and sewage waste is calculated as 960.60 t per year. Therefore, the total mass of water in the primary sewage sludge stream is 969.19 t per year and the total mass of primary sewage sludge is 1065.72 t per year. A summary of this data is presented in Table 4.

**Table 4:** Estimated data (*) to calculate the influent of sewage and wastewater to Chirnside's HTC plant in 2041

Term	Value	Unit
Wet faecal mass produced by an average person per day^99^	128	g per day
Wet faecal sludge produced by Chirnside residents in 2041*	105.12	t per year
Moisture content of faecal sludge^100^	8.17	%
Dry basis mass of wet faecal sludge*	96.53	t per year
Mass of water in wet faecal sludge*	8.59	t per year
Dry basis biomass to water ratio*	0.145	—
Total mass of dry biomass (dry food and dry faecal sludge)*	168.79	t per year
Total mass of water entering plant*	1164.07	t per year
Total mass of water added to wet faecal sludge (sewage water)*	960.60	t per year
Total mass of water in primary sewage sludge*	969.19	t per year
Total mass of primary sewage sludge (sewage water and wet faecal sludge)*	1065.72	t per year

#### 6.2.4 Chirnside: estimating the domestic thermal and electrical energy demand

To estimate the domestic thermal and electrical energy demand for the 2250 residents in Chirnside in 2041, the average domestic thermal and average domestic electricity demand per person in the UK is used. Official statistics from UK Government record the total domestic gas (thermal) and electricity consumption in 2019 as 327 419 GWh_th_ and 104 961 GWh_e_, respectively.^102^ National Statistics recorded the population of the UK in 2019 as 66 796 800.^103^ Therefore, the domestic thermal and electrical energy demand of Chirnside's 2250 residents in 2041 is estimated to be 11 029 MWh_th_ and 3536 MWh_e_, respectively (both 2 dp). This data is summarized in Table 5.

**Table 5:** Estimated data (*) to calculate the domestic thermal and electrical energy demand of Chirnside's residents in 2041

Term	Value	Unit
Total domestic thermal consumption of the UK in 2019 (ref. 102)	327 419	GWh_th_
Total domestic electrical consumption of the UK in 2019 (ref. 102)	104 961	GWh_e_
Population of the UK in 2019 (ref. 103)	66,796 800	Persons
Population of Chirnside in 2041*	2250	Persons
Domestic thermal demand of Chirnside in 2041*	11 029	MWh_th_
Domestic electrical demand of Chirnside in 2041*	3536	MWh_e_

### 6.3 The design of a hydrothermal carbonisation plant for chirnside

#### 6.3.1 Location

**Fig. 9:**
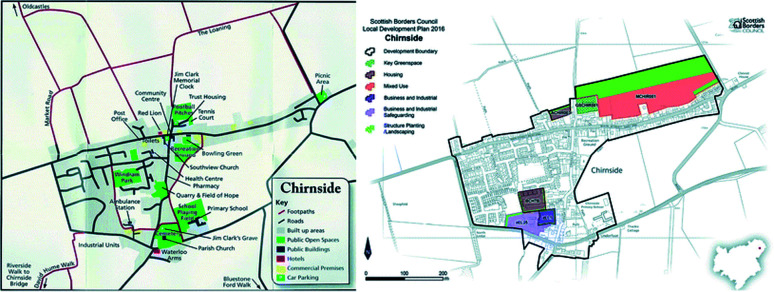
Left: map of Chirnside, Scotland. Right: map of future development plans in Chirnside.^94^

The map displayed on the left side of Fig. 9 shows the current residential layout of Chirnside. The ‘built up area’ indicated by the grey regions of the map represents local housing in the area. The right-hand map shows the boundary of current and predicted development areas, with ‘structural planting/landscaping’ and ‘mixed use’ development mainly occurring North East from the town centre. These areas have been identified in the Local Development Plan published by the Scottish Borders Council in 2016 and represents the area that would thus be unsuitable for the location of a hydrothermal carbonisation plant.

However, the area identified by the blue cross on the North-West of the left-hand map is deemed suitable for the placement of an HTC plant. This area is currently used for agricultural purpose; however, it provides an appropriate location for the plant site as it is currently uninhabited and there are no known plans for future development. This location is also convenient in terms of transporting Chirnside's biodegradable municipal and sewage waste to the site. Close proximity to the village would result in fewer emissions from biodegrade municipal waste transportation vehicles. Additionally, the capital costs for pipe-line construction, associated with the removal of sewage waste for treatment would be significantly lower when compared to a plant located several miles outside of Chirnside.

#### 6.3.2 Module sizing

Obtaining the mass of food waste (Section 6.2.2.2; 267.14 t per year) and the total mass of primary sewage sludge (Section 6.2.3.2; 1065.72 t per year) produced by Chirnside's residents annually, the total mass of waste processed at the theoretical HTC plant is calculated as 1332.86 t per year. Assuming a singular reactor module is used at the Chirnside plant, for a continuous operation of the plant for 8000 operating hours per year it must be capable of processing the combined waste at an estimated rate of 166.61 kg h^−1^ (2 dp). Estimate data is summarised in Table 6.

**Table 6:** Estimated data (*) for module sizing of the HTC plant

Term	Value	Unit
Total tonnage of waste for HTC processing in Chirnside*	1332.86	t per year
HTC plant operating hours*	8000	h per year
Continuous mass flowrate into HTC reactor*	166.61	kg h^−1^

HTC Company Ingelia have implemented their patented HTC process in UK and the Chirnside plant in Scotland is assumed to implement their technology. Ingelia's singular continuous HTC reactor has the capacity of processing 6000 tonnes of wet biomass per year.^104^ As mentioned in Section 3.1, Ingelia's reactor design can be scaled depending on the processing requirement. Therefore, a HTC plant operating in Chirnside would require a singular reactor unit at approximately 22% of the size of Ingelia's singular continuous reactor module. It is unclear if Ingelia have the capability to produce a smaller HTC reactor, or how feasible a smaller plant would be. It may be the case that it would be more feasible, from both a technology manufacturing standpoint and an economic view, to implement the larger 6000 tonne capacity reactor and look to process additional waste streams at the plant. This could include other organic fractions of municipal solid waste, domestic green and industrial agricultural wastes, and/or by collaboration with neighbouring municipalities/agricultural industries. This being said, the following sections evaluate the mass and energy balance associated with only the sewage and food waste estimated to be produced by Chirnside's 2250 residents in 2041.

#### 6.3.3 Mass balance of theoretical chirnside plant

##### 6.3.3.1 Assumptions

The following assumptions are summarized for the calculation of the mass and energy balance for the theoretical plant at Chirnside: continuous production, perfectly stirred, equipment stationary and adiabatic at the saturation pressure of water at 180 °C (10 bar), constant yields and efficiencies and no additional water being added beyond what is supplied in the sewage water (dry biomass to water ratio is constant and equal to 0.145). In addition, the moisture content and higher heating values of the faecal sludge and food waste have been taken from experimental studies, namely Danso-Boateng *et al.*^100^ and Malaťák and Dlabaja,^97^ respectively. Both of the aforementioned properties have likewise been gathered for their resulting hydrochars formed under HTC processing at 180 °C (10 bar) and 1 hour, and 190 °C for 4 hours, for the HTC of the faecal sludge and food waste, respectively. The properties of the hydrochar pellets that are produced by each waste is then “mixed” to calculate an average yield and average energy content, this implies that the properties of hydrochar are assumed to be unimpacted by the combined hydrothermal carbonisation of both the food waste and primary sewage sludge. In addition, it is assumed that residence time does not impact the hydrochar properties, which again are assumed to remain constant and equal to those taken from the literature at the specified conditions.

##### 6.3.3.2 Mass balance

**Fig. 10:**
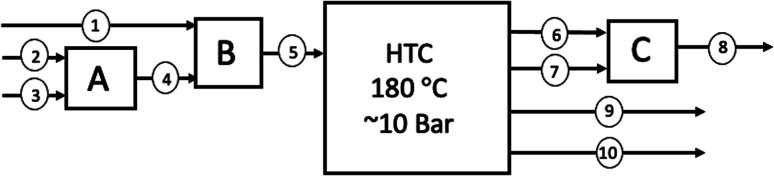
Block Flow Diagram of Chirnside's theoretical HTC plant for food and sewage waste; (1) the food waste (FW); (2) the wet faecal sludge (FS); (3) the sewage water (SW) used to transport the faecal sludge (100% water); (A) where streams 2 and 3 are mixed during transport to the plant, resulting in; (4) Primary Sewage Sludge (PSS), which is wet faecal sludge and sewage water (FS + SW); (B) where streams 1 and 4 mix before entering the HTC plant, resulting in; (5) primary sewage sludge and food waste (dry biomass/water ratio= 0.145); (6) the hydrochar produced by HTC of the faecal sludge (HC_FS_); (7) the hydrochar produced by the HTC of the food waste (HC_FW_); (C) where the properties of streams 6 and 7 are “mixed” to result in an average hydrochar of the two streams, resulting in; (8) mixed hydrochar (HC_mix_); (9) process water (PW_eff_) after separation of the solid hydrochar, and; (10) the organic fraction (ORG) which remains in either the process water as aqueous chemicals or is produced as effluent gas such as CO_2_, CO, CH_4_.

The simplified block flow diagram for the HTC processing of Chirnside's sewage and food waste is shown in Fig. 10, with a supporting mass balance presented for the process detailing the mass flowrates of the components in the respective streams in Table 8.

#### 6.3.4 Mass of hydrochar produced

Explanations of the quantifications for the annual mass flowrates for streams 1, 2, 3 and 4 are explained in Sections 6.2.2.2 and 6.2.2.3. At the Chirnside plant, it is assumed that the primary sewage sludge (stream 5) and food waste (stream 1) are mixed forming stream 5.

In order to estimate the mass of hydrochar produced at the Chirnside plant, data on the mass yields from the respective experimental studies is required. In reference to stream 6 (Table 8), Danso-Boateng *et al.*^100^ determined the faecal sludge derived-hydrochar (HC_FS_) to have a dry mass yield of 67.18% and moisture content of 4.35% when produced at the conditions specified above. Therefore, the dry mass of HC_FS_ is calculated as 64.85 t per year (2 dp), the mass of water in HC_FS_ is calculated as 2.82 t per year (2 dp) and the total mass of HC_FS_ is estimated as 67.67 t per year.

In reference to stream 7 (Table 8), Malaťák and Dlabaja^97^ determined the dry basis mass yield of experimental food waste-derived hydrochar (HC_Fw_) to be 42.30% with a moisture content of 2.76% at the aforementioned HTC conditions. Therefore, the dry mass of HC_Fw_ is calculated to be 30.57 t per year (2 dp), the mass of water in HC_FW_ is calculated as 0.84 t per year (2 dp), and the total mass of HC_FW_ is estimated as 31.41 t per year.

In this study, it is assumed that the two hydrochar producing streams (6 and 7) are “mixed” to form a mixed hydrochar stream from the two wastes. This allows for a simple estimation *in lieu* of experimental data on the hydrothermal carbonisation of these mixed wastes. However, it is important to note that the properties of a co-hydrothermal carbonisation are likely to be different to those that use this method of estimation. Nevertheless, total dry mass, total water content and total mass of HC_mix_ is calculated as 95.42 t per year, 3.66 t per year and 99.08 t per year, respectively.

The process water that remains after separation of the hydrochar is calculated as the mass of the total influx of water, less of the water that remains in the hydrochar, as 1160.41 t per year. In addition, the total dry basis mass of feedstock which does not get converted into hydrochar is calculated as 73.37 t per year. This mass is converted into soluble organics which leaves with the process water, or as gaseous effluent.

#### 6.3.5 Energy demands of the HTC plant at chirnside, and energy contained in the hydrochar produced

##### 6.3.5.1 Higher heating value of waste streams and resulting hydrochar

In order to determine if the hydrochar produced by the HTC plant at Chirnside contains enough energy to sustain the operation of the plant with a surplus for Chirnside's residents, data on the higher heating values (HHV) of the sewage and food waste hydrochars are required. Table 7 summarises the HHV's of the relevant streams, as taken from the relevant literature described in Section 6.3.3.2. The HHV's of the FS and HC_FS_ have been extrapolated from Fig. 1A and C of Danso-Boateng *et al.*,^100^ respectively, under the assumed conditions of 180 °C 1 hour reaction time. These conditions were chosen as the prediction model developed by Danso-Boateng *et al.* leads to the suggestion “that continuous-scale carbonisation can be performed at either [in reference to 180 °C for 60 min and 200 °C for 15 min or 30 min] of these operating conditions for effective carbonisation”.^100^ The HC_FS_ HHV at the specified operating conditions was determined as 17.70 MJ kg^−1^.^100^ Following this, as the HHV energy densification of 1.082 can be extrapolated from Fig. 1C under the specified operating conditions, the HHV of the FS pre-hydrothermal carbonisation is calculated as 16.36 MJ kg^−1^ (2 dp).

**Table 7:** Estimated data (*) of the energy contents of relevant streams in Fig. 10

Stream	1	2	6	7	8
Name of stream	FW	FS	HC_FS_	HC_FW_	HC_mix_
HHV (MJ kg^−1^)	4.94	16.36	17.70	28.57	21.15
Mass (t per year)	267.14	105.12	67.67	31.41	99.08
Energy content of stream (HHV) (MWh per year)	366.58	477.71	332.71	249.27	581.98

**Table 8:** Mass balance in relation to the streams presented in Fig. 10

Stream	1	2	3	4	5	6	7	8	9	10
Name of stream	FW	FS	SW	PSS	PSS + FW	HC_FS_	HC_FW_	HC_mix_	PW_eff_	ORG
Food waste (t per year)	267.14	—	—	—	267.14	—	—	—	—	—
Faecal sludge (t per year)	—	105.12	—	105.12	105.12	—	—	—	—	—
Moisture content	72.95%	8.17%	100%	90.90%	86.50%	4.35%	2.76%	3.83%	100%	—
Water (t per year)	194.88	8.59	960.60	969.19	1164.07	2.82	0.84	3.66	1160.41	—
Dry *biomass or **hydrochar (t per year)	72.26*	96.53*	—	96.53*	FW: 72.26* FS: 96.53*	64.85**	30.57**	95.42**	—	—
Hydrochar: (t per year)	—	—	—	—	—	67.67	31.41	99.08	—	—
Organics (t per year)	—		—	—	—	—	—	—	—	73.37
Total mass (t per year)	267.14	105.12	960.60	1065.72	1332.86	67.67	31.41	99.08	1160.41	73.37

Data for the FW and HC_FW_ streams has been obtained from Table 2 of Malaťák and Dlabaja following the “original sample” of “Kitchen waste” as “Raw biomass” and “Biochar”.^97^ In their study, kitchen waste (referred to as food waste from this point onwards) was subjected to hydrothermal carbonisation for 4 hours at 190 °C. The HHV of the FW in their study was determined to be 4.94 MJ kg^−1^, whereas the resulting biochar (referred to as hydrochar from this point onwards) was determined to have a HHV and a lower heating value (LHV) of 28.57 MJ kg^−1^and 26.58 MJ kg^−1^, respectively.^97^

For comparison, the energy densification (in respect to the HHV's) as a result of the hydrothermal carbonisation of faecal sludge and the food waste (including moisture content) are 1.082 and 5.783, respectively. Alternatively, the dry food waste (removing the 72.95% moisture content) resulted in a HHV of 19.46 MJ kg^−1^ and in turn results in a much lower energy densification of 1.468. Noticeably, this is still higher than the energy densification of faecal sludge.

Under the assumption that the combined HTC of both primary sewage sludge and the food waste results in a hydrochar with the combined properties of the two separate streams (6 and 7), this equates to a HHV of the mixed hydrochar in stream 8 of 21.15 MJ kg^−1^ (2 dp), and a total energy content of 581.98 MWh per year. The HHV's of the relevant streams are summarised in Table 7, along with the total energy content (HHV) of the streams reported in MWh per year.

##### 6.3.5.2 Lower heating value of hydrochar

The higher heating value of hydrochars is most often investigated and referred to in literature when assessing the potential application of the solid as a biofuel. However, when calculating the energy production potential of a hydrothermal carbonisation plant that uses hydrochar as its source of thermal and electrical energy for operation, the LHV is more appropriate to use in energy calculations as it accounts for the energy required to evaporate the moisture present in the hydrochar; otherwise known as accounting for the latent heat of vaporisation of water. Using the HHV in the energy calculations can lead to an overestimation of the energy potential and thus misinterpretation of the results. As the LHV of food waste hydrochar was given by Malaťák and Dlabaja^97^ as 26.58 MJ kg^−1^, the LHV of the faecal sludge hydrochar is estimated using eqn (1), as defined by the Federal Energy Regulatory Commission.^105^

3 LHV = HHV – 10.55(W + 9H)

where W and H represent the weight % of moisture and hydrogen in the fuel, respectively, and the HHV and LHV are in units BTU lb^−1^. The HHV of the faecal sludge hydrochar (17.70 MJ kg^−1^) is first converted to BTU lb^−1^, 7609.63 BTU lb^−1^ (2 dp). Then, the weight percentage of hydrogen (5.39%) and moisture (4.35%) in the faecal sludge hydrochar (as given by Danso-Boateng *et al.*^100^) is used to calculate the LHV of HC_FS_ as 7051.96 BTU lb^−1^ (2 dp), which equates to 16.40 MJ kg^−1^ (2 dp). The LHV of the mixed hydrochar is then estimated as a mass average of the faecal sludge and food waste hydrochars, 19.63 MJ kg^−1^. For a total mass of hydrochar of 99.08 t per year the total energy content (using the LHV of hydrochar) is calculated as 540.26 MW h per year (2 dp). A summary of these quantifications is shown in Table 9.

**Table 9:** Estimated data (*) for calculating the lower heating value

Term	Quantity	Unit
HHV of faecal sludge hydrochar^100^	17.70	MJ kg^−1^
Average HHV of hydrochar*	7609.63	BTU lb^−1^
Weight% of hydrogen in faecal sludge hydrochar^100^	5.39	%
Moisture content of faecal sludge hydrochar^100^	4.35	%
LHV of faecal sludge hydrochar (from eqn (3))*	7051.96	BTU lb^−1^
LHV of faecal sludge hydrochar*	16.40	MJ kg^−1^
Mass of faecal sludge hydrochar*	67.67	t per year
LHV of food waste^97^	26.85	MJ kg^−1^
Mass of food waste hydrochar*	31.41	t per year
Average LHV of per kg of total hydrochar*	19.63	MJ kg^−1^
Mass of total hydrochar*	99.08	t per year
Total energy content*	540.26	MW h per year

##### 6.3.5.3 Plant energy demand

To estimate the plant energy demand, this section draws on the available literature to estimate the specific thermal and electrical demands off the theoretical hydrothermal carbonisation plant in Chirnside. The specific thermal and electrical energy consumptions is the respective energy consumptions (kW h) as a function of the mass of hydrochar produced by the process (kg_HC (hydrochar)_), with a unit measure of kW h_*x*_ kg_HC_^−1^, where ‘*x*’ is representative of ‘e’ for electrical energy consumption or ‘th’ for thermal energy consumption.

Using Aspen Plus, Lucian and Fiori^106^ modelled the hydrothermal carbonisation of two waste streams -grape marc (GM) and off-specification compost (OSC)- each with different dry biomass to water ratios (DB/W) to determine the specific energy consumption of the plant. For the modelled plant, thermal power is required to both heat the biomass slurry to the HTC reaction temperature and to dry the resulting hydrochar; in the model, two methane burners are used to supply the thermal energy to the plant. In addition, the plant requires electrical energy for operating the grinder, the mixer, two pumps, a decanter, an air blower and the pelletizer, with the greatest energy demands being attributed to the pelletizer and the first pump. In their study, the specific energy consumption of the HTC plant was determined to increase as the dry biomass to water ratio decreases.^106^ That is to say that the more water and less solid mass of feedstock that is processed, the higher the thermal and electrical energy demands of the plant would be. This is expected given the greater thermal duties required to reach the elevated temperatures of a larger mass of water, and the lower mass of hydrochar that would be produced.

In order to extrapolate the specific thermal and electrical energy consumption from their study^106^ for the theoretical plant at Chirnside, the dry basis to water ratio, HTC temperature and residence time of the Chirnside plant are required. The solids loading in the pre-mixed feed (stream 5) was set at 0.145 in this study. However, the data for the two separate hydrochars obtained from two separate studies with two different operating conditions; for faecal sludge, the operating conditions were 180 °C and 1 hour residence time,^100^ and for the hydrochar produced from food waste, 190 °C and a 4 hour residence time.^97^ Therefore, as means to best represent the data from the two respective experimental hydrochar-producing papers, the operating conditions in which the specific thermal and electrical energy consumption is extrapolated from was 180 °C and a 3 hour residence time.

At a HTC temperature of 180 °C and a 3 hour residence time, Lucian and Fiori calculated a plant specific thermal energy consumption of 1.3 kWh_th_ kg_HC_^−1^ and a specific electrical energy consumption of 0.14 kWh_e_ kg_HC_^−1^ for GM, which had a DB/W ratio of 0.19. Alternatively, for OSC, the specific thermal energy consumption was 3.0 kWh_th_ kg_HC_^−1^ and the specific electrical energy consumption was 0.14 kWh_e_ kg_HC_^−1^, for a DB/W ratio of 0.07. As mentioned, the DB/W ratio for the theoretical Chirnside plant is assumed to be constant at 0.145. This is halfway between the DB/W ratio of GM and OSC. It is assumed that the same process model is used for the Chirnside plant, and that the specific thermal and electrical demand has a linear relationship with the DB/W ratio. Therefore, the specific electrical energy consumption of the Chirnside plant can be assumed to be 0.14 kWh_e_ kg_HC_^−1^, the same as both GM and OSC. Moreover, when assuming a linear relationship between the DB/W and specific thermal energy consumptions this leads to a value of 1.94 kWh_th_ kg_HC_^−1^ (2 dp). It should be noted that this is a rough estimate drawn on the extrapolation of data from two different waste streams which in themselves are different to the wastes modelled in this study. In this way, this calculation assumes that only the DB/W ratio has an impact on the thermal energy consumption of the plant and that the electrical energy consumption is not dependent on the DB/W ratio.

The estimated specific energy consumptions of the Chirnside plant can now be used to estimate the annual thermal and electrical energy consumption of the plant based on the mass of hydrochar it produces. As the plant is estimated to produce 99.08 t per year (2 dp) of hydrochar, this leads to an estimated thermal energy consumption of 192.22 kWh_th_ kg_HC_^−1^ (2 dp) and an estimated electrical energy consumption of 13.87 kWh_e_ kg_HC_^−1^ (2 dp) for the Chirnside plant. These quantifications are summarised in Table 10.

**Table 10:** Estimated data (*) to calculate the energy requirements of theoretical HTC plant

Term	Quantity	Unit
Dry biomass to water ratio of feed*	0.145	—
Specific thermal energy consumption Chirnside*	1.94	kWh_th_ kg_HC_^−1^
Specific electrical energy consumption Chirnside*	0.14	kWh_e_ kg_HC_^−1^
Mass of total hydrochar produced*	99.08	t per year
Total thermal energy consumption per year*	192.22	MWh_th_ per year
Total electrical energy consumption per year*	13.87	MWh_e_ per year

##### 6.3.5.4 Plant energy demand

In order to determine if the Chirnside plant is self-sufficient of its own production of hydrochar, the energy that is contained in the hydrochar needs to first be converted into usable thermal and electrical energy. In the literature, this step is often missing, with some studies using methane as the energy supply to the plant.^106^ However, the use of methane would make the HTC process non-renewable unless it is coupled with an anaerobic digestion plant. Therefore, to evaluate the self-sufficiency when using hydrochar as the thermal and electrical energy source for operating the HTC plant at Chirnside, it is assumed that a biomass combined heat and power (BCHP) plant is integrated with the HTC plant. In this way, any surplus thermal and electrical energy is assumed to be used by the residents of Chirnside. The following section evaluates the surplus thermal and electrical energy that can be produced by the plant after accounting for its own demands, the percentage of Chirnside's domestic energy demands that can be met and the potential revenue streams to the plant.

#### 6.4.1 Combined heat and power for thermal and electrical energy supply to Chirnside

The thermal and electrical conversion efficiencies of the BCHP plant are assumed to be 50% and 25%, respectively.^107^ At 50% thermal conversion, the total amount of thermal energy that can be produced by the BCHP plant would be 270.13 MWh_th_ per year. As the thermal energy demand of the plant is 192.22 MWh_th_ per year, the total amount of excess thermal energy that can be supplied as domestic heat to Chirnside's residents is 77.91 MWh_th_ per year. However, this is significantly lower than the total domestic thermal energy demand of Chirnside (11 029 MWh_th_ per year), thus the plant would only be able to supply 0.71% (2 dp) of this demand. This equates to the domestic thermal energy demand of approximately 16 residents. In addition, as the price of thermal energy has been quoted as €30 MWh_th_^−1^,^20^ the sales of this energy would equate to a revenue of €2337 per year (£1987 per year; 2 dp).‡‡The conversion rate taken at the time of writing was €1.00 to £0.85. However, it is important to note that not all thermal energy produced will be transferred as some will be lost to the environment or by inefficiencies in its transfer.

At 25% electrical conversion, the total amount of electrical energy that can be produced by the BCHP would be 135.07 MWh_e_ per year (2 dp). As the electrical energy demand of the plant is 13.87 MWh_e_ per year, the total amount of excess thermal energy produced by the plant that can be supplied as domestic electricity to Chirnside's residents is 121.20 MWh_e_ per year. Similarly, this is much lower than the total domestic electrical energy demand of Chirnside (3536 MWh_e_ per year), and therefore the HTC-BCHPP plant would only be able to supply 3.43% (2 dp) of this demand. This equates to the domestic electrical energy demand of approximately 77 residents and a revenue of €3394 per year (£2885 per year; 2 dp),^2^ when the price is taken as €28 MWh_e_^−1^.^20^ A summary of quantifications are presented in Table 11. This is a basic theoretical calculation and does not account for any kind of economies of scale in terms of BCHP plant capacity.

**Table 11:** Estimated data (*) for Biomass Combined Heat and Power plant results

Term	Quantity	Unit
Total energy content of hydrochar*	540.26	MWh per year

**Thermal energy**		
Thermal conversion efficiency of BCHP plant^19^	50.0	%
Thermal energy produced by BCHP plant*	270.13	MWh_th_ per year
Thermal demand of plant*	192.22	MWh_th_ per year
Thermal energy remaining to supply to Chirnside*	77.91	MWh_th_ per year
Domestic thermal energy demand of Chirnside*	11 029	MWh_th_ per year
Thermal production capacity of Chirnside's HTC plant to meet Chirnside's domestic heat demand*	0.71	%
Potential revenue from sale of thermal energy*	1987	£ per year

**Electrical energy**		
Electrical conversion efficiency of BCHP plant^19^	25.0	%
Electrical energy produced by BCHP plant*	135.07	MWh_e_ per year
Electrical demand of plant*	13.87	MWh_e_ per year
Electrical energy remaining to supply to Chirnside*	121.20	MWh_e_ per year
Electrical demand of Chirnside*	3536	MWh_e_ per year
Electrical production capacity of Chirnside's HTC plant to meet Chirnside's domestic electrical demand*	3.43	%
Potential revenue from sale of electrical energy*	2885	£ per year

#### 6.4.2 Discussion of results

It was calculated that the HTC plant would be capable of producing 99.08 t per year of hydrochar and that the HTC-BCHP plant could be both thermally and electrically self-sufficient of a portion of this hydrochar. In addition, it would be capable of supplying 0.74% and 3.44% of Chirnside's domestic thermal and electrical energy demands, respectively, with the surplus energy it is capable of producing. However, it becomes clear that this waste-to-energy process at Chirnside would not be able to meet the domestic thermal and electrical energy demand of its 2250 residents; 0.74% and 3.43% equates to fulfilling the demand of approximately 16 and 77 residents, respectively. Therefore, it can be inferred that in order to meet the total domestic electrical energy demands of Chirnside's residents, a plant with the production capacity of approximately 29 times that of the one modelled in this study would be required. Alternatively, to meet the domestic thermal demand a plant with approximately 140 times the production capacity would be required.

The coupling of a BCHP plant to convert the mass of hydrochar produced as was done in this scenario is somewhat unrealistic. This is due to the high capital cost of new BCHP plants and the low quantities of hydrochar that are expected to be produced by the plant (99.08 t per year). Thus, it could be more feasible in terms of process economics and in terms of potential energy supply to implement a HTC-CHP process capable of processing a larger quantity of organic waste. However, a life cycle analysis conducted in 2017 by Medick *et al.*^108^ that investigated the coupling of HTC both with a new BCHP plant and an existing CHP plant for the conversion of 55 000 t per year of green waste in Germany deemed all modelled scenarios as economically unfeasible at that time. That being said, it could be the case that a small-scale HTC plant coupled with an existing CHP plant as an alternative means for sewage and food waste disposal in a small town is more feasible than the large-scale HTC plants assessed in their study. However, this would only lead to minor additions to potential revenues of £2885 per year and £1987 per year for electrical and thermal energy sales, respectively. Gate fees for waste disposal through HTC could improve the economics if studied further. Lastly, the process energy balance and the mass yields of hydrochar could be improved when using a larger dry basis to water (DB/W) ratio than the one evaluated in this study (0.145). This is would also lead to a lower plant energy demand as the specific energy consumption is highly dependent on the DB/W ratio.^106^

#### 6.4.3 Conclusions

This study investigated the use of hydrothermal carbonisation as a means to process the faecal sludge and food waste of a small town in Scotland (Chirnside 2250 residents) into a solid biofuel. This study drew upon the literature for experimental data previously determined on the HTC of faecal sludge and food waste and did not perform any original experiments. The combustion of hydrochar in a biomass combined heat and power (BCHP) plant as a means to both provide the energy requirements of the HTC plant and supply some of the thermal and electrical energy demand of Chirnside's residents was assessed.

The plant modelled in this study would be capable of producing surplus thermal and electrical energy from the hydrochar it produces, once accounting for its own thermal and electrical energy demands. It has been determined that the HTC plant at Chirnside would only be capable of supplying its residents with 0.74% and 3.43% of their total domestic thermal and electrical energy demand, respectively. Therefore, the results suggest that either additional waste streams or a larger processing plant would be required to meet both energy demands, or that an alternative energy supply be used in addition to that produced from a HTC-BCHP plant. However, the economics of this model would likely benefit from integration with an existing CHP plant. This being said, based on the results it is recommended that the hydrochar that can be produced from the waste of a small town be evaluated for alternative applications; this includes as a solid biofuel for use in domestic biomass boilers, a precursor to activated carbon, a soil amendment, a catalyst and an electrode material, amongst others.

The results in this study provide a good insight to the study of HTC as a waste-to-energy process, however, in order to validate the results, experimental hydrothermal carbonisation of sewage sludge and food waste samples taken from Chirnside (or another small town) is recommended. Detailed understanding of the exact waste production figures, moisture contents, hydrochar mass yields, higher and lower heating values, real (over average) domestic thermal and electrical energy consumptions and total plant energy demands would likewise be required. In addition, the impacts of processing additional organic waste streams such as green and garden waste, the impact when adjusting the dry biomass to water ratio, modelling for economies of scale, combining waste streams from neighbouring municipalities and sensitivity and uncertainty analyses should be performed along with a full economic and environmental analysis to evaluate the feasibility of applying HTC to a small town.

## 7. Opportunity

### 7.1 Introduction to opportunities identification

The research conducted into renewable HTC technology has the main incentive of advancing humankind into a more sustainable future, specifically when comparing to the current energy production and waste disposal methods. In order to assess the opportunities that HTC technology can present in greater detail, the positive impacts that future implementation can have socially, environmentally and economically for the European Union will be explored. In addition, the opportunities presented to a small village that operates a HTC plant is explored, through application to Chirnside in the UK.

### 7.2 Policy opportunities

The European Commission, as an institution of the European Union, devised the environmental policy to outline targets for member countries to achieve. This have the main aim of protecting the health and wellbeing of EU member citizens, through environmental protection. The targets defined under the waste management section of this policy include a commitment to limit energy recovery (incineration) to non-recyclables by 2020.^109^ In order to achieve this objective, it is clear that investment into alternative waste disposal and/or energy-from-waste processes for renewable/biomass materials is required. This implies that all member countries of the EU should prioritise investment into these technologies if the target is to be achieved by 2020.

In 2019, 84% of the world's energy was derived from fossil fuel sources.^1^ The 7th Environment Action Programme (EAP) established by the European Commission aims to phasing out subsidies to environmentally harmful projects by 2020.^95^ This implies phasing out to zero of subsidies provided to fossil fuel-based projects. The EU does not publish an inventory of fossil fuel subsides and this absence of inventory reduces the ability to monitor progress. However, one study conducted with the purpose of monitoring Europe's fossil fuel subsides has made claims that the EU, through the EU budget, European public banks and related financial instruments continue to provide financial aid to the fossil fuel industry. According to the Climate Action Network^96^ an average contribution was made by the EU to the oil and gas industries of €515 million. In addition, €2 million is believed to have been provided by EU public banks for coal production in the years 2014–2016 (both inside and outside the EU). Although subsidies to fossil-fuel based industries is still occurring, the complete halt of them is unrealistic as market demand of these commodities still exists. Alongside this, the subsidies provided may be lower (phasing-out) when compared to those provided before the 7th EAP was established. The CEE Bankwatch Network have advised the European Investment Bank (EIB) to end its support for coal and non-renewable lignite power plants, as they should favour projects involving demand side energy efficiency and renewable energy sources.^97^ It is unclear if the EIB support renewable initiatives, however, the information gathered in this review (Section 4) has identified that the EU is actively investing into the development of renewable-energy technology. They do so through 7th EAP and Horizon 2020 project, which had/have a budget of €50.5 and €77 billion, respectively. This budget is significantly greater than the acclaimed subsidy amount to the oil and gas industry. More specifically, the EU has invested in multiple HTC companies besides HTC research and development projects (HTCycle, Ingelia, NEWAPP).^68,104^ The support and financial aid contributed by the EU has allowed development of HTC technology to reach a stage of commercialisation, alongside the formation of hydrochar standards which aim to increase product marketability.^104^ Therefore, despite contributions to fossil fuel industries, the EU are continuing to advance contributions towards technology that will decrease the market demand for fossil fuels. The EU's investment into the development of alternative, renewable energy solutions such as HTC today will contributes to the phasing-out of subsidies to environmentally harmful projects in the future.

### 7.3 Environmental opportunities

Hydrochar is the product of HTC with a renewable feedstock, therefore, the carbon dioxide (CO_2_) produced from combustion of this product does not contribute to a net increase in atmospheric greenhouse gas (GHG) concentrations. However, the combustion of non-renewable fossil fuels for energy generation does contribute a net increase in these emissions. Research identifies that increased atmospheric GHG emissions are the cause of many negative global impacts, such as ocean acidification, melting of polar ice caps and glaciers, rising sea levels and sea temperature, global warming and agricultural impacts, *etc.*^110^ Therefore, when comparing the two sources of energy, hydrochar is favourable when aiming to achieve sustainable future development. Alongside this, local production of hydrochar for energy applications can lead to reduction of carbon footprint (carbon emissions), as fossil fuel imports are reduced.

In addition to the opportunity for renewable energy production, HTC presents the opportunity of an effective waste disposal solution of biodegradable waste biomass when diverted from landfilling or incineration. Diversion from these practices can prevent the release of harmful emissions and in turn can prevent health risks whilst minimising the negative effects of global warming.^81^ Diversion of renewable biomass waste from incineration plants (by 2020 as defined in the EC environmental policy) to HTC plants would prevent the release of harmful and carcinogenic emissions that are known to be produced by incineration of waste. This includes production of acid gases, nitrogen oxide, heavy metals, particulates, dioxin and furans.^70^ To some degree, preventing the atmospheric release of these chemicals could be achieved *via* several control techniques within the incineration process. However, there is still risk associated with those that are not currently controlled, as well as accidental release in the case of equipment failure. Alongside this, health-effect assessments on the emissions released from incinerator plants for several hazardous compounds identified has not been completed due to emission data being ‘not readily available’.^85^ Comparatively, the production of effluent gases in a HTC process is extremely minimal (2–5%).^7^ The majority of the gaseous effluents that is produced consists mainly of CO_2_ (∼90%), with the remaining composition being a collection of hydrocarbon gases, H_2_ and CO.^91^ To date, there have been no studies into the collection, separation and utilization of the gaseous effluent produced in the HTC process. However, it has been acclaimed that there is the opportunity to produce a pure form of CO_2_, hydrocarbon gases and syngas.^111^ Therefore, a HTC plant would negate production and release of large, uncontrolled volumes of hazardous/greenhouse gases that are produced *via* incineration and/or landfilling of renewable biomass. Alongside this, there is potential to decrease the CO_2_ emissions associated with the transportation of waste if fewer transportation miles accumulate when transferring to an HTC plant over a landfill or incineration site.

The liquid product stream from the HTC process also presents an opportunity in valuable material recovery. As the effluent contains favourable amounts of beneficial organic and inorganic compounds, such as nitrogen and phosphorous, the reuse of the water on agricultural lands can enrich soils as a natural fertilizer. There are various fertilisers utilised for crop production, the choice of which depends on both the crop and the farmer. In 2013, it was estimated that the application rate of total nitrogen on the crops and grasslands in both England and Scotland was 95 kg ha^−1^ and 87 kg ha^−1^, respectively (not including phosphate, potash and sulphur).^112^ Chemical fertilizers are known to be damaging to both the environment and human health. And long-term use can change soil pH, upset beneficial microbial ecosystems, increase pests and contribute to greenhouse gas emissions. In addition, the toxic build-up of chemicals (including arsenic, cadmium and uranium) in soil escalate up the food chain into the bodies of consumers.^100^ Therefore, the production and application of a natural fertiliser, as achieved through hydrothermal carbonisation, can lead to a decrease in the application of chemical fertilizers on agricultural lands. Additionally, chemical fertilizers are primarily made from fossil fuels; the hydrogen used in the production of ammonia (Haber–Bosch process) is obtained from methane steam reforming, coal gasification or partial oxidation of oil (totalling ∼96% of worldwide hydrogen production).^113^ Therefore, natural fertiliser production *via* HTC would result in a decreased reliance on fossil fuels. In turn, application of natural fertiliser presents the prospect of progression towards sustainable future development.

**Fig. 11:**
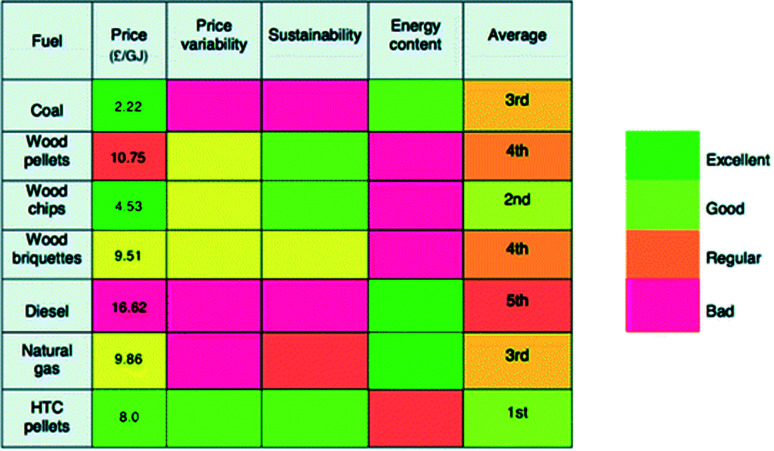
Comparison of fuel products for domestic oven use.^17^

In order to realise sustainable future development, it is necessary to compare the environmental impacts associated with the energy sources that are currently available. Fig. 11 compares the use of alternative fuel sources that can be used to power a domestic oven. When comparing the environmental impacts (sustainability) of HTC pellets and fossil-fuels (Coal, Diesel and Natural Gas), the utilisation of hydrochar is more environmentally favourable.

### 7.4 Social opportunities

The construction and operation of localised HTC plants within the European Union would create and provide long-term employment opportunities for residents in member countries. In turn, this could increase national employment. Furthermore, diversion of biomass from landfill and/or incineration plants to HTC plants can aid in the reduction of carbon dioxide emissions. Alongside mitigating the of global warming and climate change, ground level air pollution could be reduced. Exposure to carbon dioxide and other emissions released from the combustion of non-renewable fossil fuel sources have been proven to have a negative impact of human health. The ‘health bill’ associated with the combustion of coal is estimated to total €43 billion in the EU per year.^114^ Therefore, phasing-out of subsidies towards fossil-fuel based industries and directing investment into renewable energy sources, more specifically towards the development of HTC in the EU, creates social opportunities in the form of potential employment prospects and human health benefits.

### 7.5 Economic opportunities

The production of hydrochar through HTC of waste biomass can provide energy security in regions where coal, and other fossil fuels, are currently imported. The EU currently imports the majority of their coal demand from Russia, Columbia and Australia.^102^ Therefore, operating HTC plants in member countries that currently consume imported coal would provide energy security in the event of interruption/termination of supply. Alongside this, fossil-fuel reserves continue to deplete with time whilst population and energy demand continue to grow. Therefore, a countries energy security *via* renewable technologies will contribute to greater economic stability in the future.

Although the capital and operational expenditure associated with an HTC plant can be high due to the technology being relatively new to the market, the implementation of HTC has associated several monetary gains. As previously described, revenue can be generated from the direct sales of hydrochar pellets (coal), application of these for electricity generation, activated carbon production (used as supercapacitor electrode material). In turn, the profitability of the company depends on the quality of hydrochar produced, the final application, and the capital and operational expenditures. Moreover, the process water can be sold for fertilization of crops and gate fees could be collected from the disposal of biomass (depending on the market in which HTC is applied). A profitable HTC company can improve the local economy if the plant is owned, constructed and operated by local companies. Alongside this, an HTC can increase potential employment prospects and therefore improve the local economy. Additionally, exportation (of hydrochar) can improve the gross domestic product (GDP).^115^

In order to assess the economic opportunity HTC presents, the costs associated with the consumption of common household fuel products and HTC pellets (hydrochar) must be compared. In addition to a comparison of sustainability, Fig. 11 shows how the price, price variability and energy content of the fuels have been ranked for domestic oven use. As shown, the combination of the rankings from these categories forms an average ranking position, which places hydrochar pellets in 1st place. Coal is shown to have the cheapest fuel price at £2.22 per Giga Joule (GJ), whereas hydrochar pellets are priced at £8 per GJ. Despite this, comparison of fuel price to the alternative fuel types presented in Fig. 11 demonstrates that hydrochar pellets are competitive within the fuel product market, due to their relatively low cost. Alongside the low-cost evaluation, the pellets present a valuable economic opportunity to those countries who produce it, as their price variability is the highest. This is due to pellet price being independent of both political and economic policies, varying only slightly with energy content, which is dependent on the biomass feedstock. Stable prices of hydrochar pellets can prove a beneficial opportunity to economy, as stable commodity prices contribute to a country achieving high levels of economic activity and employment.^116^

### 7.6. Conclusion on the opportunities HTC presents

The implementation of both industrial and commercial HTC plants throughout the EU is a viable solution to achieving the target to phase-out environmentally harmful subsidies by 2020, as defined in the environmental policy.^117^ Through funding businesses established within the field of HTC for energy production, *via* initiatives established by the EU such as Horizon 2020, the reliance on fossil-fuel energy production in the EU would be reduced. Increased investment and/or re-direction of subsidies to research and development within HTC will not only aid in achieving the phasing-out of subsidies to fossil-fuel industries, but also aid in phasing-out of fossil fuel reliance. With this comes the opportunity to combat global warming, climate change and the negative impacts associated. In addition, optimising HTC as an alternative biomass waste disposal method will lead to diversion of biodegradable municipal waste from landfills (the least preferable option of waste disposal as defined in the Landfill Directive (1999/31/EC)^65^) and/or incineration plants, in which combustion of high moisture feeds is inefficient and is to be limited to only non-recyclables by 2020.^109^ Diversion of waste biomass from both of these practices can also prevent the release of harmful (carcinogenic) compounds and greenhouse gas emissions (carbon dioxide and methane). The stable prices of pellets (due to unpolitical pricing) and competitively priced hydrocar can contribute to achieving high levels of economic activity and employment.^116^ Alongside this, hydrochar pellets are competitively priced when compared to other fuel sources.^104^ Member countries who import coal can benefit from localised/industrial plants as hydrochar pellets are capable of replacing coal, benefiting from energy security. However, the EU's switch from fossil fuels to renewable sources is proving to be a slow, transitional process. Overall, HTC plants present many valuable opportunities for the UK and all the countries of the EU which, with continued research and investment, would benefit the most as we aim towards a more sustainable state of living in line with the UNs sustainable development targets.

## 8. Challenges in HTC technology

Alongside the many positive opportunities that hydrothermal carbonisation can present, the identification of potential challenges to overcome is necessary to secure its implementation in future projects. The sections below identify some of the more concerning challenges currently faced in HTC, alongside any potential solutions that are being/can be further explored to overcome them.

### 8.1 New technology

As explored in Section 4, HTC plants are currently being operated worldwide. Although interest in its application is increasing, it should be noted that HTC technology is new and in the early stages of implementation. The National Sludge Strategy, conducted by Scottish Waters, classified the pyrolysis of wastewater sludge as ‘relatively high risk’, as they are ‘commercially unproven technologies with planning and procurement time equal to or greater than an incineration plant’. They also stated that ‘They [includes gasification] typically have high capital and operating costs’.^118^ However, since these statements were published in 2006, numerous research projects and advancements have been conducted for the development and industrial application of HTC technology. In addition, high capital and operational costs can be expected from the construction of any new technology-based processing plant. Therefore, in order to achieve efficient and sustainable development, a re-evaluation of the waste processing techniques outlined in the 2006 National Sludge Strategy is due if implementation of HTC is to be encouraged.

In recent years, there has been significant amounts of funding in support of the research and development into HTC technology. However, there are other energy-from-waste processes that have already established themselves within the market and are rapidly expanding. One of which is Anaerobic Digestion AD: the treatment of biodegradable (food) waste and sewage waste using microbes for biogas (methane) production. In 2014, the UK recorded over 100 plants in operation which rapidly grew to 640 plants in 2018.^119^ Investments made towards AD development and application is contributing to sustainable future development. Thus, the plentiful investments into an established business model can make it challenging for a HTC company to compete with. Based on this, HTC has great potential to rival AD due to the following comparative advantages.

**Fig. 12:**
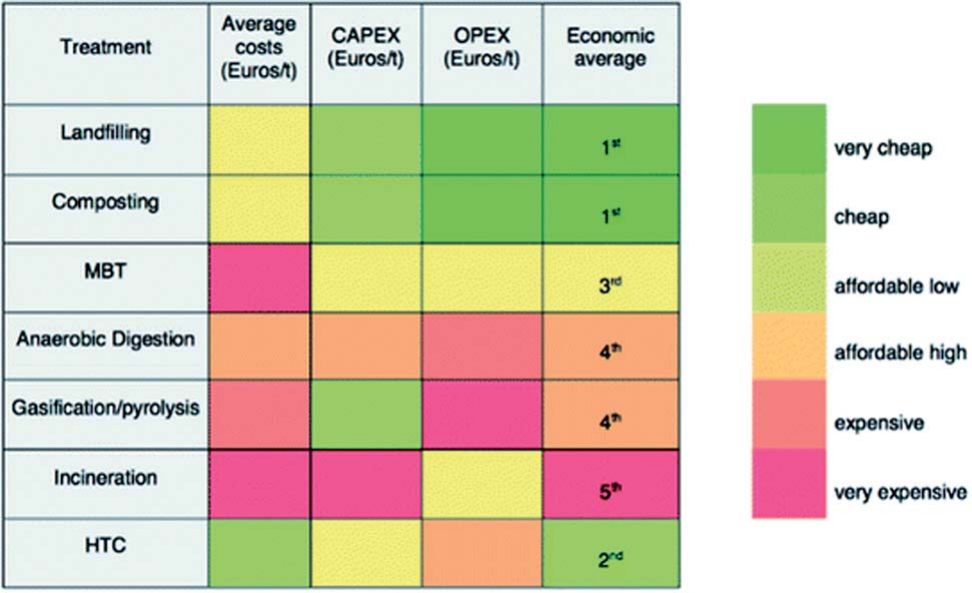
Economic comparison of current waste disposal techniques.^17^

In comparison to a solid fuel produced by HTC, the enzymes in AD produce biofuel in the form of a gaseous product. Storage and transport of a gaseous fuel can, in some instances, be challenging, costly and pose a greater risk due to storage equipment requirements, high pressures and potential leaks. Moreover, the AD of municipal biodegradable waste must be complete free of any food waste packaging to avoid operational challenges. Whereas, HTC is capable of depolymerising plastics if not completely removed from the feedstock. In addition, AD requires large land requirements whereas HTC can process large masses of waste over a small plant footprint.^35^ What is more, the cost of AD is greater than the cost associated with HTC (Fig. 12). However, the most noticeable difference between these two technologies is efficiencies: the efficiency of HTC is approximately 5 times greater than that of AD, and almost double the carbon efficiency.^120^ To conclude, when debating between investment into AD and HTC development, the main point to consider is the less efficient, more expensive production of a gaseous fuel, or the cheaper, more efficient production of a solid fuel.

This being said investigations into the coupling of the two technologies to form AD-HTC hybrids can solve the problem of by-product use for the other. Investigations include utilising AD technology to produce biogas from the upstream HTC reactors process water.^121^ However, one HTC company claims to produce clean process water through their HTC technology/process, thus eliminating the requirement of AD for the treatment of process water.^120^ The reverse operation has likewise been investigated, where HTC is used to process the digestate remaining after AD.^122^ Reza *et al.* found that processing AD digestate through HTC results in a greater amount of energy per 1 kg of raw biomass, which is 20% and 60% more than that of HTC alone and AD alone, respectively.^15^ Therefore applications of HTC in conjunction with existing AD plants may grow over the following decades as companies previously invested in AD aim for greater energy outputs. Investment by AD operators can thus aid in overcoming the challenge of technology marketability.

Another energy-from-waste treatment method in which HTC must compete with is incineration of waste; the UK Government's Department for Environment Food & Rural Affairs recorded that 83 incinerator plants were operational in 2014.^79^ However, HTC has been proven to be more energetically viable than incineration of wet biomass for moisture contents greater than 10%.^24^ Concluding that albeit HTC is a new technology, the process should be the new choice to avert such inefficiencies experienced in both anaerobic digestion and the incineration of high moisture biomass feeds.

As HTC is a new technology, there are still many unknowns about the exact performance details of the process. This arises due to each lignocellulose biomass feed capable of being processed through HTC having a different percentage of hemicellulose, cellulose and lignin. This allows for kinetic modelling to be completed; however, it is specific to the type of feedstock which it is completed for.^123^ Even then, exact reaction mechanisms are unknown and there will be discrepancies in the properties of the hydrochar produced. To some degree, not knowing exact process details can correlate to a client's insecurity towards the technology. However, to combat any concern potential, HTC companies such as Antaco (UK) and Ingelia (Spain) that have patented their process should provide support and reassurance to potential clients. For example, Antaco offers a wide range of services that include organic waste assessment, site assessment, feasibility and costings.^74^ And, Ingelia have stated that they will ‘establish cooperation, framework and joint venture agreements with international partners to support the deployment of [our] HTC plants all over the world’.^81^ The method of a joint venture business entity with the HTC specialist demonstrates the company's collaboration method and confidence in the ability of their process to perform. In turn, this provides clients and investors with assurance in the new technology. Additionally, NEWAPP is producing a standardised quality database for experimental data recorded on the different feedstocks with the aim of providing assurance and encouraging marketability.

### 8.2 Logistics

Implementing a HTC plant alongside a process that currently generates a biomass rich waste stream leads to semi-straightforward calculations for the logistics and reactor module sizes. However, the logistics for feed transportation from homeowner to plant can become challenging. In terms of both economic and environmental costs, it would be inefficient to collect the municipal waste from each household *via* heavy duty vehicles every day of the week. Therefore, the logistics of waste transport and the associated cost are dependent on the operational capacity of the plant and the average household's municipal waste production over a week (as to prevent adverse side effects from storing food waste). From this, efficient waste collection logistics can be achieved through computer simulations that account for these dependencies. This would allow for the most efficient routes and collection days to be calculated with easy revision each year. This method may be costly to initiate, however, most towns within the UK have already established waste disposal logistics which can be analysed to adapt to the location of the HTC plant. Implementing heavy-duty vehicles for waste collection in areas which do not currently operate waste collection can have negative environmental impacts due to the release of carbon dioxide emissions. Thus, emissions could be reduced if the distance travelled to a HTC plant is shorter in comparison to the established waste processing site. Moreover, the use of heavy-duty vehicles may be reduced and even completely eliminated if food waste disposal systems (sink shredder) are applied to new (and old) households. This would mean that shredded food waste would flow in the wastewater stream (in the sewage system) towards the HTC plant.

The transport of sewage sludge in the UK is achieved through an underground waste water sewage system which is transported towards the ‘sewage works’ or ‘wastewater treatment’ plant.^124^ Therefore, diversion of the underground sewage system that encourages flow towards a HTC plant can be challenging and costly to achieve. Additionally, wastewater systems typically contain around 0.1% of solid matter.^88^ Therefore, preliminary and primary treatment methods would need to be located close to the HTC plant in order to separate the organic fraction (unless a separate toilet sewage system is constructed). Therefore, location of the HTC plant in accordance to the established sewage system and WWT plant containing both primary and pre-treatment methods would be most beneficial when processing waste waters. This demonstrates the practicality of operating AD-HTC hybrid plants for wastewater treatment as described in Section 8.1. The HTC plant would be capable of processing the by-product of AD (digestate) and the AD could process (purify) the HTC process water.

As demonstrated, choosing the right logistic methods for a HTC town plant, alongside implementing the chosen method, can be challenging and time consuming, especially for a HTC plant with a continuous onsite feed stream that requires minimal logistic consideration. However, by efficiently completing the above, the biodegradable municipal and sewage waste produced by the residents of a town in the UK can provide just over a third of its entire energy demand.

### 8.3 Economics

#### 8.3.1 Capital and operational expenditure

The associated expenditures for construction of any new plant or processing facility can be expected to be high, with HTC being no exception. A HTC plant capable of processing 22 000 tonnes of biomass has been estimated to cost €7.8 million until the payback period of 5.5 years is reached.^104^ Alternatively, a 20 000 tonne plant has been estimated to cost £10 million until the payback period of 10 years is reached.^106^ Thus, an economic comparison study of various waste disposal methods has ranked the HTC business model in 2nd place overall. This comparison method is shown in Fig. 12, where current waste disposal methods have been ranked on their average costs, capital expenditure (CAPEX) and operating expenditure (OPEX). HTC is the highest ranked energy-from-waste disposal process as it only ranks below the average cost associated with landfilling and composting. Neither of these methods produces energy and therefore they produce little to no profits. Comparing the energy-from-waste processes in Fig. 12 shows that the CAPEX of HTC is ranked as ‘affordable low’, whereas anaerobic digestion and incineration are ranked ‘expensive’ and ‘very expensive’, respectively. The OPEX associated with HTC is ranked as ‘expensive’ which can be associated to the cost of high pressure and temperature reactor coupled with the new type of technology when compared to older waste treatment methods. Comparatively, a correlation between the investment cost into an incineration plant and the processing tonnage has been established by Waste to Energy International; for a 20 000 tonne plant the investment cost is estimated at £16.5 million.^125^

#### 8.3.2 Hydrochar pricing

Due to the small number of operational HTC plants, the current supply situation causes the hydrochar pellets to be less competitively priced (£8 per GJ) when compared to the prices of coal and woodchips (£2.22 and £4.53 per GJ, respectively). Nevertheless, future prices of competing fossil-based fuels can be expected to increase due to the oil supply policy change in producer countries, increased upstream production costs, decreased reserves and stricter environmental policies.^126^ Besides, increasing the investments towards the development of HTC technology can decrease the CAPEX and OPEX of future HTC projects. In turn, operators can choose to reduce the price of hydrochar or improve their profitability.

#### 8.3.4 Biomass feed

Dependant on the industry in which it is implemented, a challenge in the concern of potential investors could arise from a predictable depletion of their biomass feedstock. For example, operation of a unit that is to process Brussel sprout or orange-peel waste would mean that the feedstock quantities are highly dependent on the given season. Other factors that can lead to an unpredicted depletion of biomass feedstock include climate change, crop failure and changing farming strategies. Some of the factors contributing to feedstock fluctuations are beyond the control of unit operators and should be considered by potential investors. Thus, the HTC plant should be flexible, having a wide variety of biomass feedstock to be processed if there was ever a decline in feedstock supply. Therefore, if intended feedstock were to become scarce for any circumstance, alternative biomass feeds can be (pre)sourced for processing to ensure revenue and investment security.

A challenge concerned with the varying ‘pumpability’ of the feed slurry is that the lower the moisture content of the biomass entering the reactor, the more difficult the pumping operation becomes. However, this challenge can be circumvented by adding a recycle stream of the process water to the feed, as shown in Fig. 11 and 12.^104^

#### 8.3.5 Product variability and complications

Overall mass and energy balance calculations over a HTC plant used to estimate hydrochar production, quality and heating value (*etc.*) have margins for error, even based on experimental data. This is due to the heterogeneity of the biomass feed composition coupled with the unknown details of the reaction mechanisms. However, the increasing interest into HTC by academics, investors and governing bodies are propelling the research required to reduce the error and uncertainty. To understand the processing of different feeds under different conditions and minimise the error in HTC feasibility calculations, a standardised quality criteria of the products from alternative feedstock is currently being developed by the European Biomass Industry Association (EUBIA) in association with the International Organisation for Standardisation (ISO).^104^ Once these standards have been established and are industrially recognised, consistency in the quality of hydrochar produced can be achieved and marketability can be improved.

Inorganic materials such as stones, pieces of metal, dust and sand have reportedly been found in the product streams. When present in the biomass feedstock, unlike the organic compounds, they are not destroyed during the process reactions. Their presence can lead to penalties on the process energy balance, as energy can be wasted from the unit trying to process and heating the inert material. However, experimental research conducted on a waste stream with a high amount of inorganic material (>20%) demonstrated that HTC can still proceed smoothly. This means that HTC is a robust process and that technical problems due to chemical composition are of minor importance.^104^ However, it should be noted that the presence of any large solid particles can also damage valves and process pumps. Thus, any suppliers of biomass feed to a HTC plant should be notified to prevent contamination from large inorganic materials as separation is difficult and inviable.

The contamination of the biomass feed with any heavy metals such as mercury, lead and chromium will lead to their persisting presence in the product process water and hydrochar. These can be introduced from printed paper or batteries entering the process. Due to the high toxic risk factor associated with heavy metals present in the feed even at extremely low concentrations, the extraction from the products is paramount. Special attention should be paid on the extraction of heavy metals from any process water that is to have agricultural applications, such as a fertiliser, or if the solid hydrochar is to be alternatively applied as a soil conditioner. Hydrochar contaminated with heavy metals with applications for fuel can still be valorised energetically but must be done under controlled conditions.^104^ Another potential contaminant of the HTC process water is that of persistent organic pollutants (POPs) which are resistant to environmental degradation. Very little is known about their presence in the HTC process water but to ensure they are not. Preliminary tests should be conducted on new sources of biomass feed.

## 9. Conclusion and perspectives

HTC is an effective method for the treatment of biodegradable municipal waste and sewage waste. Compared with the traditional landfill and incineration method, it can greatly decrease the emission of harmful gases. In addition, the energy produced is renewable and is generated with no net CO_2_ production, making it sustainable. The major product, hydrochar, can also bring good profit. The assessment of HTC plant in a small village (Chirnside) further confirms the promising application of this technology. Both social and economic benefit could be expected. However, there are still some challenges for HTC replacing the current process:

(1) As a new technology, there are many unknown mechanisms in HTC process. In addition, it has to compete with current waste disposal methods, as well as other renewable energy technologies.

(2) The logistics system for HTC can be both time consuming and costly.

(3) The associated expenditures for construction of HTC can be expected to be high. The price of hydrochar is competitive, however; the price is currently higher than the price of coal in equivalent Joules of energy.

(4) There might be some uncertainty in the resulting quality of hydrochar due to the complexity of different biomass sources used and possible contamination of the biomass feed.

In light of these challenges, HTC may be better applied to centralised waste biomass producing facilities. In this way, logistics will be reduced, and expenditure for the conversion is more justifiable and in line with the concepts of the circular economy and industrial ecology models. In addition, the most suitable and feasible application of different waste-derived hydrochars should be holistically evaluated between the following applications to determine the most feasible application; centralised production of energy (BCHP), decentralised distribution of solid fuel (use in domestic biomass boilers), activated carbon production, electrode/battery material, as a catalyst, or as a soil conditioner. Lastly, prediction of the hydrochars characteristics under different HTC conditions would allow for an optimal application to be pre-determined and could thus increase deployability of hydrothermal carbonisation across different industries worldwide.

## Conflicts of interest

There are no conflicts to declare.

## Supplementary Material
